# Evolutionary adaptation from hydrolytic to oxygenolytic catalysis at the α/β-hydrolase fold†

**DOI:** 10.1039/d3sc03044j

**Published:** 2023-09-18

**Authors:** Soi Bui, Sara Gil-Guerrero, Peter van der Linden, Philippe Carpentier, Matteo Ceccarelli, Pablo G. Jambrina, Roberto A. Steiner

**Affiliations:** a Randall Centre for Cell and Molecular Biophysics, King's College London London SE1 1UL UK roberto.steiner@kcl.ac.uk; b Departamento de Química Física, University of Salamanca Salamanca 37008 Spain pjambrina@usal.es; c European Synchrotron Radiation Facility (ESRF), Partnership for Soft Condensed Matter (PSCM) 71 Avenue des Martyrs Grenoble 38000 France; d European Synchrotron Radiation Facility (ESRF) 71 Avenue des Martyrs 38043 Grenoble France; e Université Grenoble Alpes, CNRS, CEA, Interdisciplinary Research Institute of Grenoble (IRIG), Laboratoire Chimie et Biologie des Métaux (LCBM) UMR 5249 17 Avenue des Martyrs 38054 Grenoble France; f Department of Physics, University of Cagliari Monserrato 09042 Italy matteo.ceccarelli@dsf.unica.it; g IOM-CNR Unità di Cagliari, Cittadella Universitaria Monserrato 09042 Italy; h Department of Biomedical Sciences, University of Padova Italy roberto.steiner@unipd.it

## Abstract

Protein fold adaptation to novel enzymatic reactions is a fundamental evolutionary process. Cofactor-independent oxygenases degrading *N*-heteroaromatic substrates belong to the α/β-hydrolase (ABH) fold superfamily that typically does not catalyze oxygenation reactions. Here, we have integrated crystallographic analyses under normoxic and hyperoxic conditions with molecular dynamics and quantum mechanical calculations to investigate its prototypic 1-*H*-3-hydroxy-4-oxoquinaldine 2,4-dioxygenase (HOD) member. O_2_ localization to the “oxyanion hole”, where catalysis occurs, is an unfavorable event and the direct competition between dioxygen and water for this site is modulated by the “nucleophilic elbow” residue. A hydrophobic pocket that overlaps with the organic substrate binding site can act as a proximal dioxygen reservoir. Freeze-trap pressurization allowed the structure of the ternary complex with a substrate analogue and O_2_ bound at the oxyanion hole to be determined. Theoretical calculations reveal that O_2_ orientation is coupled to the charge of the bound organic ligand. When 1-*H*-3-hydroxy-4-oxoquinaldine is uncharged, O_2_ binds with its molecular axis along the ligand's C2–C4 direction in full agreement with the crystal structure. Substrate activation triggered by deprotonation of its 3-OH group by the His-Asp dyad, rotates O_2_ by approximately 60°. This geometry maximizes the charge transfer between the substrate and O_2_, thus weakening the double bond of the latter. Electron density transfer to the O_2_(π*) orbital promotes the formation of the peroxide intermediate *via* intersystem crossing that is rate-determining. Our work provides a detailed picture of how evolution has repurposed the ABH-fold architecture and its simple catalytic machinery to accomplish metal-independent oxygenation.

## Abstract 2

We report how the common α/β-hydrolase architecture and its simple catalytic machinery have been adapted to accomplish spin-restricted metal-independent oxygenation.

## Graphical abstract

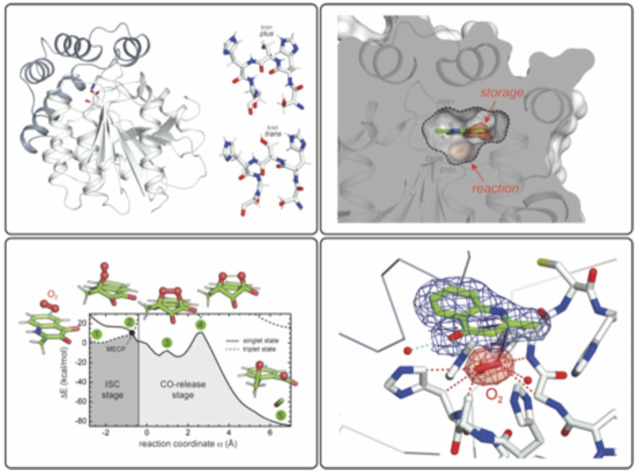

## Introduction

The switch from a reducing to an oxidative atmosphere during our planet's history spawned the emergence of many O_2_-dependent enzymes, several of which evolved from pre-existing classes, whose original functions are unrelated to dioxygen chemistry.^1–4^ The α/β-hydrolase (ABH) fold is a common and versatile protein architecture found in all three domains of life. The comprehensive ESTHER (ESTerase, α/β-Hydrolase Enzymes and Relatives) database lists more than 70,000 family members that typically share little sequence similarity but display a common fold and catalytic machinery.^5^ Their core structure is an eight-stranded β-sheet surrounded by α-helices often decorated with additional structural elements, generally referred to as cap or lid domains, that confer functional variation (Fig. S1†).^6,7^ Mechanistically, most ABH-fold members rely on the conserved nucleophile–histidine–acidic residue proton-relay system of serine hydrolases. In keeping with this, the majority of ABHs are lipases, proteases, esterases, thioesterases, dehalogenases, and epoxide hydrolases that use H_2_O as the co-substrate. However, this is not universal and other members include haloperoxidases, lyases, and even oxygenases that employ H_2_O_2_, HCN, or O_2_, respectively, for catalysis.^8^ This underscores the functional malleability of the ABH fold and its simple catalytic triad.

**Fig. 1:**
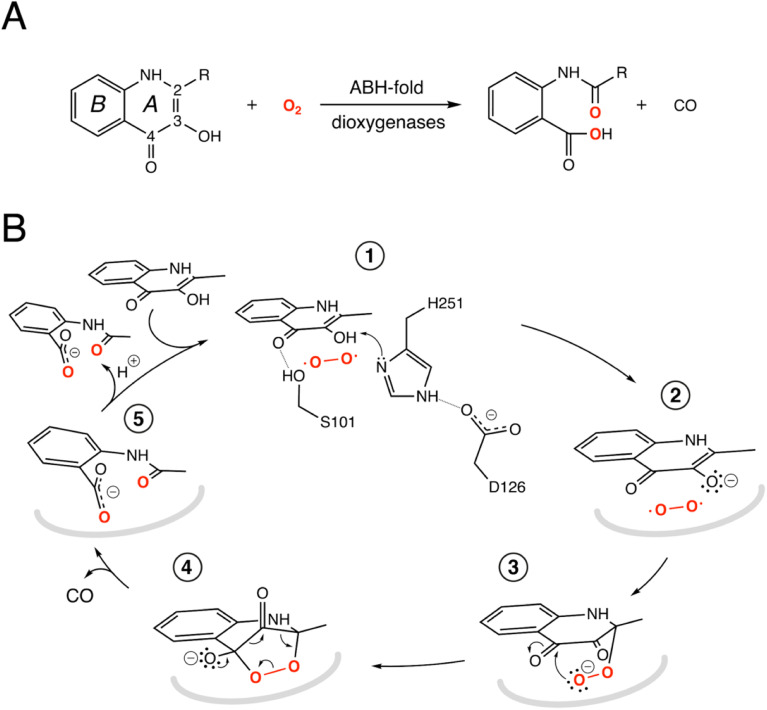
ABH-fold cofactor-indepdendent dioxygenase reaction and mechanism. (A) Scheme of the reaction catalyzed by the bacterial cofactor-independent ABH-fold dioxygenases. HOD primarily catalyzes conversion of 1-*H*-3-hydroxy-4-oxoquinaldine (R=CH_3_, QND) to *N*-acetylanthranilate (NAA). In the reaction, the A heterocycle is disrupted by the formation of carbon monoxide as a by-product. The compound 2-methyl-quinolin-4(1*H*)-one (MQO) used in this work features –H instead of –OH at position 3; (B) reaction mechanism as exemplified by HOD. The structure of the anaerobic HOD–QND complex revealed that the 3-OH group of QND is deprotonated by the H251-D126 pair of the ‘nucleophile–histidine–acidic’ ABH-fold triad (steps 1 and 2). S101 at the sharp structural turn known as the ‘nucleophilic elbow’ further stabilizes the substrate at the active site. In some ABH-fold dioxygenases an alanine residue replaces the serine at the ‘nucleophilic elbow’. The reaction is then assumed to proceed *via* the peroxide intermediates (3 and 4) to the formation of the product NAA (5). Hydrogen bonds are represented by dotted lines. In reaction steps (2–5) the protein environment is shown in a simplified manner as a grey curved line.

*Arthrobacter nitroguajacolicus* Rü61a 1-*H*-3-hydroxy-4-oxoquinaldine 2,4-dioxygenase (HOD) and *Pseudomonas putida* 33/1 1-*H*-3-hydroxy-4-oxoquinoline 2,4-dioxygenase (QDO) were the first dioxygenases discovered to belong to the ABH-fold family.^9,10^ They catalyze the O_2_-dependent breakdown of *N*-heteroaromatic quinolone-type substrates with concomitant carbon monoxide production (Fig. 1A). Recently, AqdC1 and AqdC2 from *R. erythropolis*, and AqdC from *M. abscessus* subsp*. abscessus*, have been also identified as ABH-fold dioxygenases that catalyze the same reaction,^11,12^ and the family of quinolone-type degrading ABH-fold dioxygenases has been significantly expanded by bioinformatic analyses with the identification of more than 150 *bona fide* new members, nine of which have been validated experimentally.^13^ Atypical for oxygenases, these enzymes promote the activation of the triplet ground-state O_2_ molecule without the aid of metal centers or external organic co-factors.^14^ Kinetic measurements indicate that their reaction mechanism relies on a fast initial step, during which the substrates' 3-hydroxyl group is deprotonated by the His-Asp subset of the triad, thus promoting its activation for molecular oxygen attack (step 1, Fig. 1B), whilst the nucleophile is not essential.^4,15,16^ Consistent with this, in various organisms, a serine to alanine replacement is observed for the ‘nucleophile’ residue (Fig. S2†). The role of the His-Asp dyad as a general base is supported by the crystal structures of HOD and AqdC that have been solved in mechanistically relevant states.^4,17^ The dispensability of the nucleophile is in stark contrast with that observed for most ABHs for which the nucleophile is required for the formation of the mandatory covalent acyl-enzyme intermediate.

Cofactor-independent ABH-fold oxygenases are intriguing from a mechanistic viewpoint as the spin-forbidden oxygenation reaction is enabled by a minimalistic catalytic toolbox. Theoretical investigations have put forward different hypotheses for the mechanism that allows the quantum chemical hurdle of the direct reaction between the singlet-state substrate anion and the triplet-state O_2_ to be overcome.^18,19^ According to one QM/MM study the rate limiting step of the reaction is the addition of O_2_ to C2 of QND leading to the formation of peroxide (steps 2–3, Fig. 1B) that has been suggested to occur on the triplet-state potential energy surface with a 17 kcal mol^−1^ barrier, followed by an intersystem crossing leading to a singlet state.^18^ Another study instead suggested that the triplet-state peroxide intermediate is unlikely to play a role in the mechanism and that catalysis could proceed *via* a direct electron transfer from the QND anion to O_2,_ followed by radical recombination yielding the peroxide intermediate.^19^ Once the peroxide 3 is formed, subsequent ring closure (step 4) followed by release of CO and formation of the product (step 5) are uncontroversial.

The most elusive and interesting aspects of cofactor-independent oxygenation at the ABH-fold are those that involve molecular oxygen for which no direct structural information is currently available. In this work, we have addressed this gap using an integrated computational and experimental framework. This approach has allowed a complete understanding of the reaction mechanism to be obtained, providing a rationale for how this versatile protein architecture and its simple catalytic machinery tuned for hydrolytic reactions has been successfully redeployed during evolution to carry out O_2_-dependent catalysis.

## Results and discussion

### HOD displays an O_2_ pocket that partly overlaps with that of the organic substrate.

**Fig. 2:**
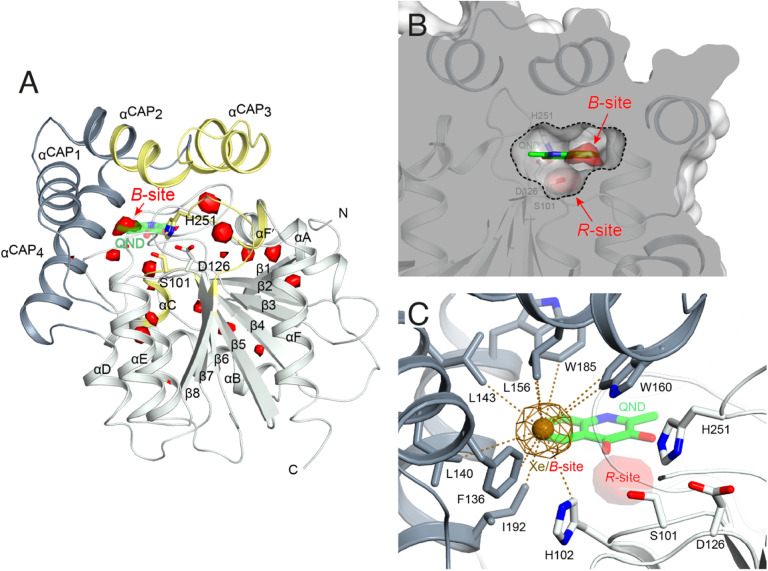
Dioxygen binding sites mapped by MD simulations and xenon pressurization. (A) Cartoon representation of HOD (core and cap domains in light and dark grey, respectively) with high-probability O_2_-sites (isosurface representation in red) as revealed by MD simulations. Structural regions whose dynamics is reduced by dioxygen are highlighted in yellow. Most O_2_-sites are temporary pockets generated by protein dynamics within the core domain. One site (B-site) is much more accessible and overlaps with the location occupied by the B-ring of the QND substrate in the E–S complex. The latter is shown for reference as a stick representation with carbon, nitrogen and oxygen atoms colored green, blue and red, respectively. Residues of the catalytic triad are also shown as sticks; (B) sliced-surface back-view (roughly rotated by 180° around the vertical axis compared to the view in A) showing a cut-through of the active site cavity in the E–S complex (PDB code 2WJ4) with its largest section highlighted by a dotted line. The B-site lies within the flat horizontal portion of the active site that hosts the QND substrate. Underneath the substrate's A-ring heterocycle, the active site is shaped into a basin. The R-site (R for reactive) that measures approximately 15 Å^3^, positioned in front of the S101 sidechain, is expected to host O_2_ during catalysis; (C) active site of HOD following xenon pressurization. A Xe atom, shown as a gold sphere with its 2*mF*_o_-*DF*_c_ map at the +1.0*σ* level as a chicken-wire representation, localizes at the B-site. Stabilizing sidechains are shown as sticks with atoms within 5.0 Å of Xe highlighted by dotted lines. Residues of the catalytic triad are also shown. All residues are colored according to the domain to which they belong. The bound QND substrate and the R-site underneath it are shown for reference.

To identify possible dioxygen pockets within HOD we initially performed molecular dynamics (MD) simulations. Two independent replica simulations, each 1 μs-long, were carried out for water-solvated HOD in the presence of dioxygen (ten O_2_ molecules in a box of approximately 74 × 74 × 74 Å^3^). Identical simulations were also performed without explicit dioxygen. Hereafter, we will refer to simulations with and without explicit O_2_ as OXY-MD and WAT-MD, respectively. In all runs, we observed only minor deviations from the starting crystallographic model (PDB code 2WM2) as expected from the stable ABH fold (Fig. S3†). In OXY-MD runs, we sampled O_2_ positions every 50 ps by mapping the locations most frequently visited sites on this time scale (Fig. 2A). These tend to be confined to the core domain and are typically transient pockets afforded by sidechain movements. Amongst the top sites there is however also a portion of the active site (B-site in Fig. 2A) that is substantially more accessible. This location overlaps with the binding site of the natural substrate, specifically that of its hydrophobic B-ring portion (Fig. 1A). We also compared root-mean-square fluctuations (r.m.s.f.) of the Cα coordinates from the average structures in OXY-MD and WAT-MD runs (Fig. S4†), a measure of the structural changes during the simulations. This suggests that O_2_ tends to reduce the mobility of the β5-αC ‘nucleophilic elbow’ centered at S101, the portion of the cap domain overhanging it, and the β8-αF′ C-terminal loop hosting the catalytic H251 residue. These regions (highlighted in yellow in Fig. 2A) define the interface between the core and cap domains where the active site is located. The basin-shaped portion of the active site that is expected to host O_2_ for its attack on the activated substrate (Fig. 2B) is not amongst the most frequented locations on the 50 ps time scale. This O_2_ ‘reactive-site’ (R-site) was identified in the crystal structure of the anaerobic HOD–QND complex as a ∼15 Å^3^ cavity opening in front of S101 underneath the substrate's A-ring^4^ and corresponds topologically to the ‘oxyanion hole’ that stabilizes the negatively charged tetrahedral transition state in hydrolytic reactions.^7,20^

We next employed xenon pressurization in the crystal state to probe O_2_ sites experimentally. Electron-rich Xe is quite easily detected by crystallographic methods and has been successfully used to visualize hydrophobic O_2_ binding sites in proteins.^21–23^ Pressurization of HOD crystals in a quartz capillary at room-temperature under a constant 30 bar Xe atmosphere allowed us to measure X-ray data at 2.9 Å using synchrotron radiation (Table S1†). Fourier difference density maps revealed strong peaks consistent with xenon binding at the B-site (as it overlaps with the substrate's B-ring in the HOD–QND complex) of all four HOD molecules present in the asymmetric unit (Fig. 2C). Modelling these peaks as water molecules resulted in significant positive residual density post-refinement, supporting the notion that Xe occupies this site (Fig. S5A†). Anomalous maps are also consistent with this assignment (Fig. S5B†). Occupancy refinement of Xe atoms gives values in the 40–60% range. Xe is stabilized at the B-site by the hydrophobic environment of the H102, F136, L140, L143, L156, W160, W185, I192 side chains with the closest atoms 3.8–5.0 Å from the gas atom (Fig. 2C). Although we performed our experiment at room temperature, which compared to cryo-conditions, allows for enhanced protein, mobility we have not identified additional Xe binding sites in our maps.

Overall, experiments and simulations agree that the ABH-fold HOD dioxygenase features an O_2_ pocket (B-site) within the active site at a location that overlaps with the most hydrophobic portion of its aromatic substrate.

### S101 at the nucleophilic elbow modulates O_2_/H_2_O stability at the R-site

HOD's active site shape suggests that O_2_ must be located at the R-site to react with the bound substrate (Fig. 2B). Unlike the hydrophobic B-site, the R-site is polar and crystallographic studies have shown that a water molecule is often bound at this position with variable occupancy.^4,16^ In the presence of NaCl at high concentration the R-site also stabilizes a chloride ion.^4^ Halide and dioxygen binding sites have been shown to be shared in other O_2_-dependent enzymes.^24–27^

**Fig. 3:**
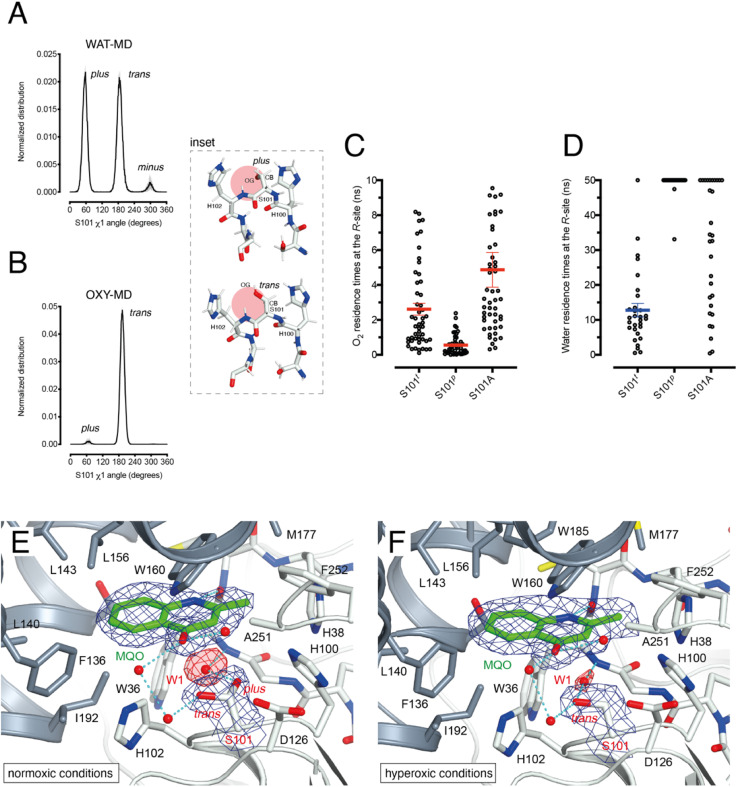
S101 as the O_2_/H_2_O modulator at the R-site. (A) Normalized distribution of *χ*1 (N-CA-CB-OG) angles for S101 in MD simulations carried out for HOD without explicit dioxygen. S101 rotamers are defined as: *plus* (*χ*1 = +60°), *trans* (*χ*1 = 180°), and *minus* (*χ*1 = −60° = 300°). Values are the average of two independent MD replicas each 1 μs-long sampled every 50 ps. Standard deviation is shown as light grey bars; (B) same as A in the presence of explicit dioxygen. The inset provides snapshots during the simulations with the R-site represented by the red circle. The CB-OG bond lines the R-site for the *trans* rotamer whilst it roughly points into it for the *plus* rotamer; (C) distribution of residence times for an O_2_ molecule at the R-site of the HOD–QND complex with S101 restrained to the *trans* (S101^*t*^) and *plus* (S101^*p*^) rotamers or with S101 replaced by an alanine (S101A). A total of 50 independent MD simulations were performed for each system. Mean and s.e.m. values are highlighted; (D) like C but for a water molecule at the R-site. A total of 30 independent MD simulations were performed for each system with a cutoff time of 50 ns. For S101^*t*^, S101^*p*^, and S101A water was observed leaving the R-site within the cutoff (29/30), (2/30), and (20/30) times, respectively. Mean and s.e.m. values for S101^*t*^ are highlighted; (E and F) active site of the HOD^H251A^–MQO complex under normoxic (E) or hyperoxic (F) conditions. Electron density maps (2*mF*_o_-*DF*_c_) are shown at the +1.0*σ* level as a chicken-wire representation for MQO, and the water molecule at the R-site (W1) and S101. The latter displays a mixture of *plus* (40%)/*trans* (60%) rotamers under normoxic conditions whilst only the *trans* rotamer is observed under hyperoxic conditions. Electron density for W1 is displayed in red for clarity. The H-bond network involving MQO, W1 and S101 is shown as cyan dotted lines.

As dioxygen must be in strong competition with H_2_O for the R-site and considering the proximity of S101, we wondered whether this residue might play a role in modulating the O_2_/H_2_O preference for this location. Serine residues are characterized by three main rotational conformers (rotamers) for their (N-CA-CB-OG1) *χ*1 torsion angle defined as *plus* (*χ*1 = +60°), *trans* (*χ*1 = 180°), and *minus* (*χ*1 = −60°). Statistical analysis shows that *trans* and *plus* are the least and most frequently observed, respectively, particularly when this residue is not part of either α-helices or β-strands.^28^ We analyzed S101 rotamer preferences in our 1 μs-long substrate-free WAT-MD and OXY-MD simulations and found that O_2_ shifts the distribution from bimodal, in which both *plus* (S101^*p*^) and *trans* (S101^*t*^) rotamers are sampled with equal frequency (Fig. 3A), to unimodal in which S101^*t*^ dominates (Fig. 3B). The *minus* rotamer is never frequently populated. Structurally, the transition from S101^*p*^ to S101^*t*^ causes the CB-OG bond to reorient itself, switching from the direction pointing directly toward the R-site to one ‘running’ tangentially to it following the backbone (Fig. 3 inset).

Next, we turned our attention to the enzyme–substrate (E–S) complex. As expected, a 1 μs-long MD run starting from the crystallographic HOD-QND structure^4^ revealed only limited deviations from the experimental model (Fig. S6†). To assess if a possible correlation exists between the S101 rotamer preference and O_2_ stability at the R-site we then performed multiple independent simulations in which a single O_2_ molecule was positioned at this location underneath the substrate and monitored its residence times with S101 restrained either to its *trans* or *plus* rotamer. A total of 50 simulations were carried out for each rotamer. We find that the *trans* rotamer stabilizes O_2_ at the R-site better than the *plus* rotamer with mean residence times of 2.61 ± 0.34 ns and 0.55 ± 0.08 ns, respectively (Fig. 3C). Also, a wide time distribution is observed when S101 is restrained to the *trans* rotamer, including times up to 8 ns, whilst times are short and tightly distributed for the *plus* rotamer. Alanine substitution (S101A) results in a behavior like S101^*t*^ (mean ± s.e.m. of 3.95 ± 0.40 ns) albeit with generally longer residence times. We have also estimated residence times (*k*_off_^−1^) from the numerical fitting of the cumulative time distribution of individual events that is expected to follow Poisson statistics. This procedure gives similar *k*_off_^−1^(O_2_) values (Fig. S7†).

Next, we carried out 30 more independent simulations and performed a similar analysis for a H_2_O molecule located at the R-site (Fig. 3D). As expected, water is retained at this location longer than O_2_. However, whilst for *plus*-restrained S101, water was observed to leave the R-site only twice within a cutoff of 50 ns (6.7% escapes, with individual residence times of 33.1 and 47.5 ns), in the case of *trans*-restrained S101 water stability is significantly reduced (96.6% escapes, mean ± s.e.m. of 13 ± 2 ns). In the case of the S101A substitution, H_2_O stability is somewhat intermediate between the behavior promoted by the two S101 rotamers (67% escapes) with a large time distribution. Numerical fitting gives a *k*_off_^−1^(H_2_O) value of approximately 15 ns for S101^*t*^ (Fig. S7†), while it is greater than 50 ns for both S101^*t*^ and S101A.

Overall, our analysis suggests a role for S101 as a modulator of O_2_/H_2_O stability at the R-site. Transient O_2_ binding within the HOD architecture favors the switch to the *trans* rotamer which, in turn, promotes both O_2_ stabilization and H_2_O destabilization at the R-site. The S101A replacement at the nucleophilic elbow appears to be a useful strategy to further stabilize O_2_ at the R-site at the cost, however, of less effective water clearing.

### Normoxic and hyperoxic HOD^H251A^–MQO structures validate S101 as an O_2_/H_2_O modulator.

To visualize O_2_ at the R-site, we turned to pressurization experiments in the crystal state using the ‘soak-and-freeze’ method that allows crystal cryocooling under pressure.^29^ This is a significant advantage over methods that require pressure release before cryocooling as it minimizes the escape of gas molecules that often have high *k*_off_ rates.

Initially, we employed the near-inactive HOD^H251A^ variant that under normoxic conditions affords the visualization of a stable HOD^H251A^–QND complex.^16^ However, O_2_-pressurization of the complex (40 bar O_2_ for 2 minutes) leads to the complete conversion of the bound QND into the product (see the ESI and Fig. S8†). This clearly indicates that in the crystal state under hyperoxic conditions, O_2_ can reach the R-site in the preformed E–S complex. To prevent turnover, we next synthesized the substrate analogue 2-methyl-quinolin-4(1*H*)-one (MQO). MQO, a non-reactive molecule structurally closest to the natural substrate, in which the hydroxyl group at position 3 is replaced by the –H substituent. As crystals of HOD^H251A^ typically diffract better than those of wild-type HOD we solved the X-ray structure of this variant in complex MQO under normoxic and hyperoxic conditions at the 2.0 Å and 2.1 Å, resolution, respectively (Table S1†). MQO binds in the active site similarly to QND indicating that the lack of the 3OH group does not result in substantial changes. MQO is held in place by a single H-bond between its NH group and the carbonyl oxygen of W36 at 2.8 Å. Several residues contributed both by the core domain (Gly35, Trp36, Cys37, His38, His100, Ser101, His102, Gly103, and Phe252) and the cap domain (Phe136, Leu140, Leu143, Leu156, Trp160, Met177, Trp185, Ser188, Gly189, and Ile192) further stabilize the ligand with hydrophobic interactions (Fig. 3E). A water molecule (W1) is present at full occupancy at the R-site underneath the A-ring of MQO 2.8 Å from its molecular plane. It is stabilized by an interaction with the main chain amide of W36 and the side chain of S101 which is observed in double conformation in both molecules present in the a.u. with *trans* and *plus* rotamers refining at occupancies of 0.60 and 0.40, respectively. W1 is H-bonded to S101^*p*^ 2.7 Å from its OG atom, whilst S101^*t*^ (OG) is more than 3.8 Å away.

We next inspected electron density maps following the O_2_-pressurization experiment (40 bar O_2_ for 2 minutes) (Fig. 3F). These do not reveal changes that we could positively ascribe to O_2_ bound at the R-site. Instead of the elongated electron-rich density observed for bound dioxygen in other systems,^29,30^ O_2_-pressurization led to a decrease in electron density at the R-site compared to that under normoxic conditions. Occupancy refinement of a water molecule at this location gives values of 0.67 and 0.48 in the two independent molecules present in the a.u. Remarkably, S101 shifts completely to the *trans* rotamer (*χ*1 values of 180.08 and 180.66°) with no indication of the alternative *plus* rotamer observed under normoxic conditions.

Overall, these observations indicate that O_2_ most likely interferes with H_2_O binding at the R-site, and although disorder negates its positive identification, this nevertheless leads to the shift of S101 to the *trans* rotamer that, in agreement with the simulations, appears to be positively correlated with the presence of dioxygen at the R-site.

### O_2_ access to the R-site in the pre-formed E–S complex

As *in crystallo* O_2_ pressurization promoted turnover of the HOD^H251A^–QND complex we again employed MD simulations to gain an understanding of possible access O_2_ routes to the R-site in the pre-formed E–S complex. We performed a total of 80 independent 200 ns standard MD runs either in the presence of ten O_2_ molecules placed in a box of approximately 74 × 74 × 74 Å^3^ (OXY10-S-MD runs) or using a single O_2_ molecule placed initially near W37 and constrained within a sphere of 17 Å radius from S101 (OXY1-S-MD runs). The latter condition decreases the sampling space while still allowing O_2_ to diffuse outside the protein.

**Fig. 4:**
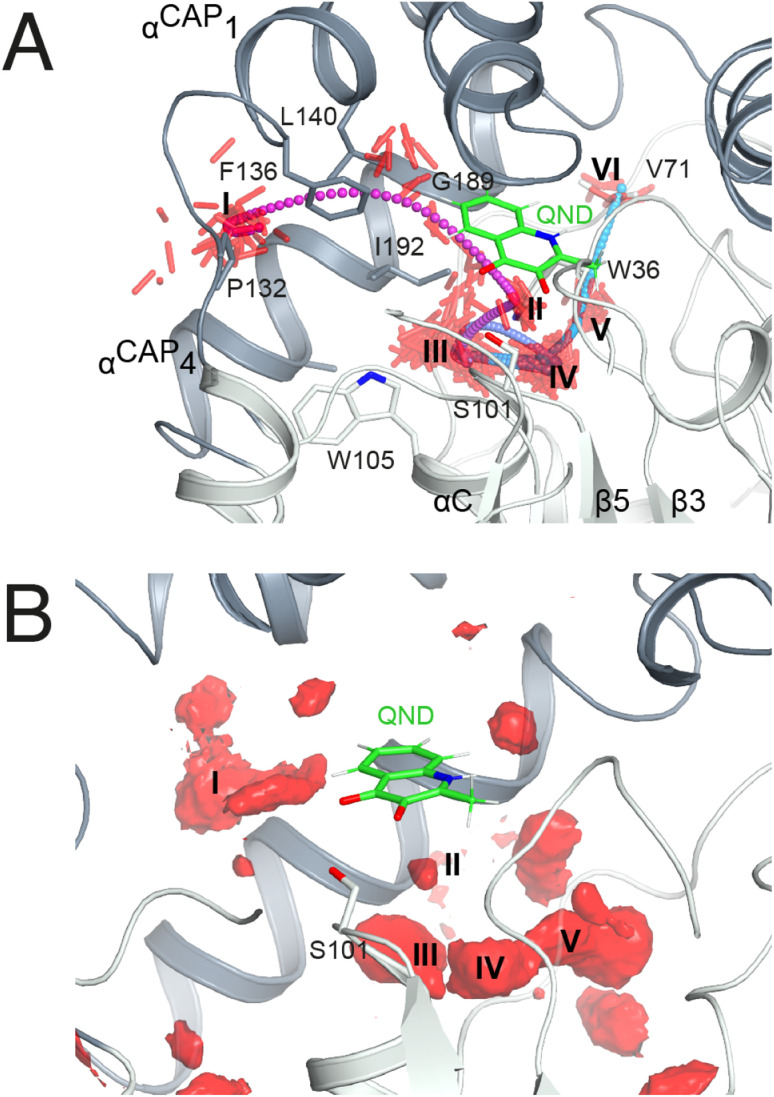
O_2_ access to the R-site in the E–S complex. (A) Dioxygen trajectory extracted from a standard MD simulation. Roman numbers indicate clusters of dioxygen molecules, shown as red sticks, sampled every 50 ps. The O_2_ trajectory, highlighted by small spheres colored using a cyan-magenta gradient (entry–exit), shows that dioxygen reaches the R-site (cluster II) from cluster I, and exits the ABH-fold *via* clusters (III–VI) as described in the main text. (B) Regions of high O_2_ occupancy probability (isosurface representation in red) obtained reconstructing the free energy in the Cartesian space from metadynamics simulations. Roman numbers indicate the same clusters of panel A.

The simulations reveal that O_2_ entry at the R-site is not a frequent event. Out of the 80 standard MD simulations we observed spontaneous O_2_ entry at the R-site only once in either OXY10-S-MD or OXY1-S-MD runs. These events occurred when S101 was restrained to its *trans* rotamer. O_2_ access to the R-site followed the same trajectory in both productive runs (Fig. 4A). Dioxygen entered the ABH-fold near P132 (cluster I in Fig. 4A) and reached the R-site (cluster II) taking advantage of a hydrophobic path lined by residues belonging to *α*_CAP1_ (F136 and L140) and *α*_CAP3_ (I192 and G189). O_2_ then escaped from the R-site highlighting clusters III and IV near the nucleophilic elbow. These were recurrently visited prior to reaching clusters V and VI close to W36 and V71, respectively. Cluster V matches very well a high-probability O_2_ pocket seen in MD simulations in the absence of the substrate.

In a second set of calculations, we employed biased metadynamics simulations with a single O_2_ molecule placed in the bulk and free to visit any location inside the box (OXY1-S-metaD runs). The use of a bias (see the Methods section) allows the sampling of the entire 3D Cartesian space with simulations restricted to 1.8 μs each to be accelerated. OXY1-S-metaD runs highlight the same clusters I–V seen in the standard simulations in addition to a few additional clusters (Fig. 4B). Energy calculations confirm a lower barrier for O_2_ entry when S101 is in the *trans* rotamer (4.1 ± 1.1 kcal mol^−1^) compared to the *plus* rotamer (6.5 ± 1.5 kcal mol^−1^). This corresponds roughly to a five-fold increased probability in the case of the former.

### Visualization of O_2_ at the R-site

As the simulations suggested that the S101A substitution decreases *k*_off_(O_2_), we then solved the structure of HOD^S101A^ in a complex with MQO under normoxic and hyperoxic conditions at 2.0 Å resolution (Table S1†). The HOD^S101A^ variant was also considered interesting as the AqdC ABH-fold dioxygenase that catalyzes a chemical reaction identical to HOD naturally possesses an alanine instead of a serine at the nucleophilic elbow (hydrophobic elbow).

**Fig. 5:**
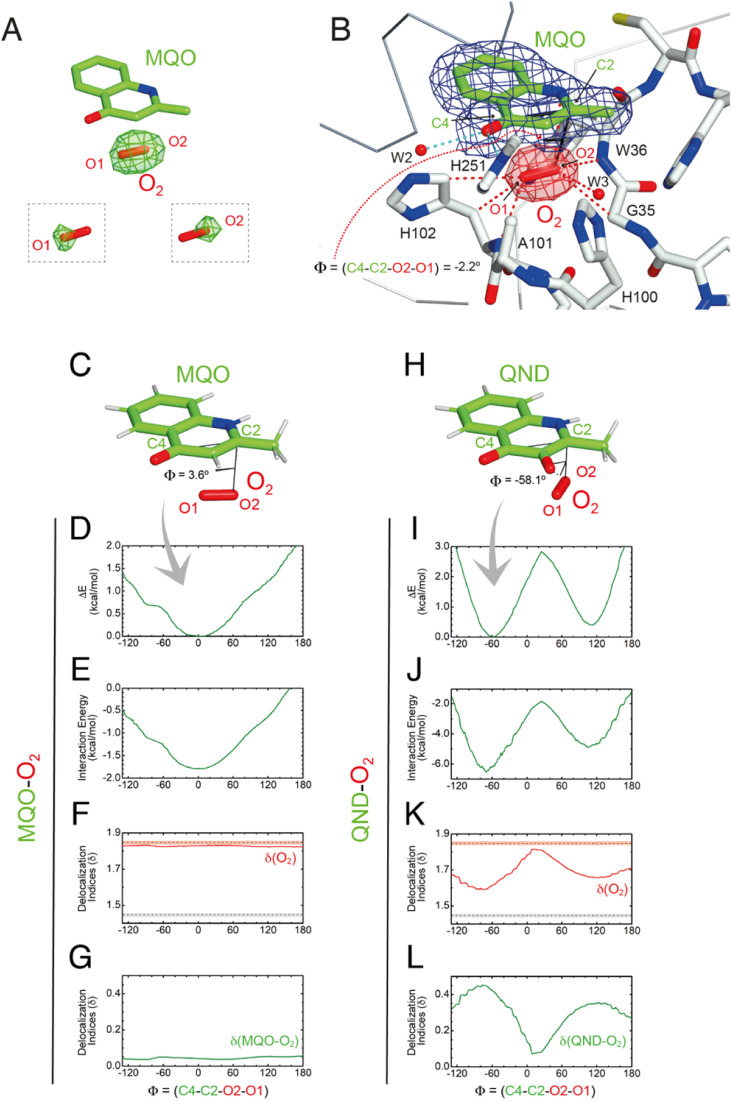
Visualization of dioxygen at the R-site and QM validation. (A) Following O_2_ pressurization (40 bar for 2 minutes) of the HOD^S101A^–MQO complex, *mF*_o_-*DF*_c_ Fourier difference maps at 2.0 Å-resolution (in green at the +3.0*σ* contour level) reveal a strong elongated peak at the R-site underneath the MQO ligand that is consistent with dioxygen. O_2_ from the refined model is shown for reference as a red stick. The insets show the *mF*_o_-*DF*_c_ electron density map (+3*σ*) with O1 and O2 atoms selectively removed from crystallographic refinement. (B) 2*mF*_o_-*DF*_c_ electron density of the HOD^S101A^–MQO–O_2_ complex. Electron density maps are shown at the +1.0*σ* level as a chicken-wire representation for MQO (blue) and O_2_ at the R-site. The latter is displayed in red for clarity. The dihedral angle *Φ* = (C4–C2–O2–O1) in the refined crystallographic model is −2.2°. (C–L) QM geometry restrained optimization and delocalization indices (*δ*) for the MQO–O_2_ (C–G) and QND–O_2_ (H–L) complexes as a function of the dihedral angle *Φ*. (C and H) Stick representations of the minimized structures, (D and I) total energy, (E and J) interaction energy-only, (F and K) *δ* for O_2_, and (G) sum of the pair-*δ* between the ligand and O_2_ for the MQO–O_2_ and the QND–O_2_ complexes, respectively. In (H and J) *δ* values for isolated O_2_ (dashed red line) and the aromatic C–C bond in benzene (dashed grey line) are given for reference. Experiment and theory are in excellent agreement that the MQO–O_2_ complex displays an energy minimum close to *Φ* = 0°.

MQO binds to HOD^S101A^ as observed with HOD^H251A^. Although the S101A replacement causes the loss of the H-bond between S101(OG) and MQO(O4) this is compensated by the interaction with H251(NE2) resulting in an essentially identical binding mode. Under normoxic conditions, electron density at the R-site of HOD^S101A^ is consistent with the presence of a water molecule (Fig. S9†). However, in contrast with what was observed for the HOD^H251A^–MQO complex, following O_2_-pressurization (40 bar O_2_ for 2 minutes) Fourier difference maps revealed a strong elongated peak at the R-site of one of the two HOD^S101A^ chains in the asymmetric unit (Fig. 5A). This peak ranked third overall for height (+7.3*σ*) with the first two corresponding to sodium ions. Modelling of this peak as an O_2_ molecule did not result in negative difference density, and occupancy refinement converged to unity with atomic displacement parameters for both oxygen atoms consistent with those of the surrounding atoms (average *B*-value MQO = 21.3 Å^2^, *B*-value O_2_(O1) = 20.5 Å^2^ and *B*-value O_2_(O2) = 19.7 Å^2^). Moreover, omit maps for the individual oxygen atoms produced difference Fourier peaks, supporting the assignment as O_2_ (insets of Fig. 5A). The equivalent peak in the other protein chain was less prominent and more spherical in shape. We have modelled this peak also as O_2_ and occupancy refinement converged at 0.80 with an average *B*-value of 30.3 Å^2^. This value is marginally higher than that of the MQO molecule in its proximity (average *B*-value = 21.9 Å^2^) suggesting higher rotational disorder.

Using the fully occupied O_2_ molecule as a reference, we find that dioxygen binds underneath the A-ring of MQO approximately parallel to its plane at an average distance of 3.2 Å (Fig. 5B). The ligand's atoms closest to O_2_(O2) and O_2_(O1) are C2 at 3.1 Å and C4 at 3.4 Å, respectively, and dioxygen further interacts with the main chain amide groups of W36 and H102 as well as with the side chain of the latter residue. Next to the R-site, a water molecule (W3) is also present at a H-bond distance from O1 (2.7 Å) stabilized by the side chain of H100 that is flipped compared to the HOD^H251A^ variant. W3 and H100 flipping are also seen under normoxic conditions in two of the four molecules present in the a.u. The O_2_ molecular axis is roughly parallel to the MQO(C2–C4) direction as quantified by the dihedral angle *Φ*(C4–C2–O2–O1) of −2.2°. Thus, in the HOD^S101A^–MQO–O_2_ ternary complex, dioxygen appears to preferentially adopt an orientation that mimics that of the endoperoxide during the catalytic cycle (step 4 in Fig. 1B).

### The geometry and electronic properties of the ligand–O_2_ complex depend on the ligand's charge.

To seek further insight into the geometry and electronic properties of the QND–O_2_ and MQO–O_2_ complexes, we performed *ab initio* quantum mechanics (QM) calculations. Initially, we considered two systems constituted only by the ligand (either QND or MQO) and O_2_. As QND is deprotonated by the His-Asp dyad, we assumed that the QND–O_2_ system bears a single negative charge whilst MQO–O_2_ is neutral. In these calculations, we restrained the sum of the (C2–O1) and (C4–O2) distances to the crystallographic values. However, around the equilibrium geometry, our results are insensitive to small changes in the restraint value (Fig. S10†).

The total energy of the complexes as a function of *Φ* is shown in Fig. 5D and I. Despite the structural similarity between QND and MQO, the QM calculations revealed that the orientation of O_2_ with respect to the ligand is different. Whilst in the MQO–O_2_ complex, dioxygen orients its axis along the MQO(C2–C4) direction with a dihedral angle *Φ* of 3.6° (Fig. 5C) in excellent agreement with our crystallographic structure, in the QND–O_2_ complex, O_2_ is aligned with the QND(N1–C3) bond at an angle of about −58.1° (Fig. 5H). We observe that an essentially identical angular dependence is observed for the “interaction energy”-only component of the total energy (Fig. 5E and J), indicating that molecular distortions are not important in defining the equilibrium geometry of these systems. Energy decomposition analysis allowed the further investigation of the contribution of the different terms (Pauli repulsion, charge transfer, electrostatic, dispersion, and polarization) to the interaction energy (Fig. S11†).^31,32^ For the MQO–O_2_ complex, the most stable orientation is that which minimizes the Pauli repulsive energy between the electronic clouds of MQO and O_2_, that is, in this case, associated with π–π stacking. Differently, for the QND–O_2_ complex, the equilibrium geometry is that which maximizes the charge-transfer from the QND anion to O_2_. We hypothesized that the difference in the relative O_2_ orientation between the two complexes is caused by the negative charge of QND. This was confirmed by calculations for the protonated (neutral) QNDH–O_2_ complex, that closely mirror those obtained for MQO–O_2_ (Fig. S12†).

Next, we analysed the electron density shared between the ligand and O_2_ using delocalization indices (*δ*), which are intimately related to bond order.^33^ We calculated these values as a function of *Φ* for dioxygen *δ*(O1–O2), and for the sum of all the pair-*δ* values between the ligand and O_2_, *δ*(QND–O_2_) and *δ*(MQO–O_2_). For the MQO–O_2_ complex, we find that independently of *Φ*, *δ*(MQO–O_2_) is close to zero whilst *δ*(O1–O2) is close to the value for isolated O_2_, *δ*(O1–O2) = 1.84 (Fig. 5F and K). This indicates that there is essentially no electron density transfer between MQO and O_2_. For the QND–O_2_ complex, both *δ*(QND–O_2_) and *δ*(O1–O2) are no longer independent of *Φ*. Moreover, at the equilibrium geometry, where charge-transfer is maximized, *δ*(QND–O_2_) has a maximum at ∼0.35 while *δ*(O1–O2) has a minimum at ∼1.67, which is intermediate between the value for isolated O_2_ and that of an aromatic C–C bond (Fig. 5G and L). In this complex therefore electron density is shared between QND and O_2_ that results from the electron transfer from the π cloud of QND to the π* orbital of O_2_, decreasing its bond strength.

To test the effect of the protein matrix we have repeated the same analysis above using a QM/MM model in which the QM region includes the substrate, O_2_ and key active site residues (Fig. S13A and E†). In these calculations that explicitly include the active site environment, no restraints were employed. These more sophisticated calculations do not alter our previous conclusions and the results are entirely consistent with that obtained for the isolated complexes (Fig. S13B and F†). We note, however, that for MQO–O_2_, the energy difference associated with O_2_ rotation is smaller, with a barrier height lower than 0.5 kcal mol^−1^, which could be easily overcome at thermal energies. This flat energy profile therefore allows rotational averaging which likely contributes to an increase in the sphericity of O_2_ electron density at the R-site as observed in one of the two active sites. For the QND–O_2_ complex, the barrier is about 2 kcal mol^−1^, like that obtained from the isolated complex. Analysis of the delocalization indices also produced results like those obtained for the isolated complexes (Fig. S13C, D, G and H†).

### Reaction mechanism

**Fig. 6:**
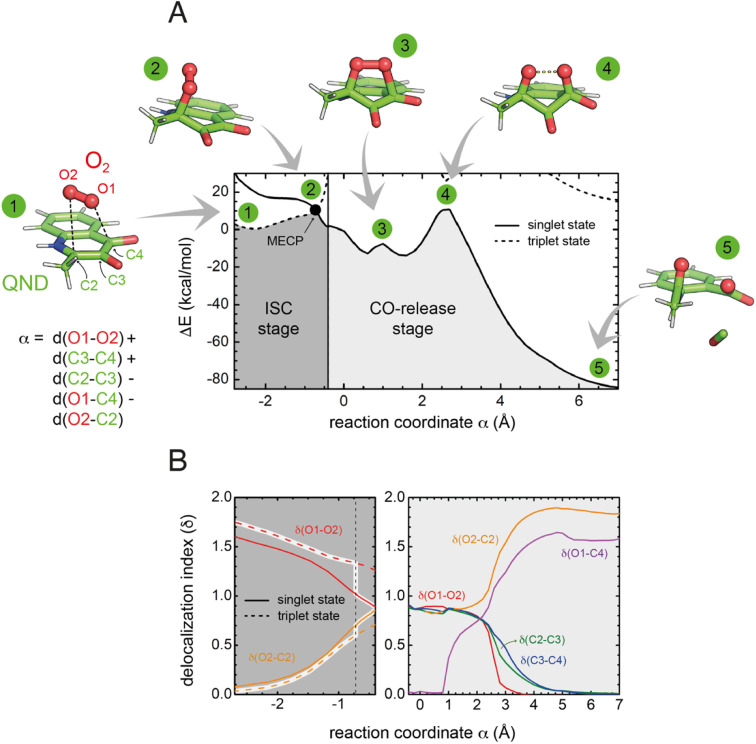
Energetics and charge transfer along the reaction path. (A) Energy profile calculated at the M06-2X/6-31+G(d,p) level of theory as a function of the reaction coordinate *α*. The lowest energy singlet and triplet states are shown as continuous and dashed lines, respectively. Numbered key stages of the reaction are visualized as ball-and-stick representations. In all of them, the protein is not drawn for simplicity. The vertical line (at *α* = −0.4) distinguishes the two reaction stages. The left-hand region defines the intersystem crossing (ISC) stage, in which the system switches from the triplet to the singlet state leading to the formation of the peroxide through the minimum energy crossing point (MECP, highlighted by a black dot). The CO-release stage on the singlet potential energy surface is strongly exothermic. (B) Evolution of the delocalization indices (*δ*). The left panel shows *δ* indices for the ISC stage where only *δ*(O1–O2) and *δ*(O2–C2) change significantly. The DIs are shown for the triplet (dashed lines) and singlet (solid line) states, and those corresponding to the most stable state are highlighted. The vertical dashed line represents the position of the MECP. Differences between *δ* values for the singlet and triplet states at the MECP indicate that ISC is associated with a significant transfer of electronic density from the substrate to O_2_. In the right-hand side panel, *δ* indices are shown only for the singlet state, as the triplet state is very high in energy.

To investigate the reaction mechanism, we calculated the QM/MM energy profile along the reaction coordinate *α* (Fig. 6A). The reaction can be conveniently rationalized as composed of two stages: (i) an intersystem crossing (ISC) stage in which the system ‘hops’ from the triplet to the singlet state with the formation of C2-peroxide (2) and (ii) the CO-release stage, in which the peroxide breaks down *via* endoperoxide (3 and 4) leading to products (5). For the ISC stage, the energy profile was calculated as a function of the energy difference between the singlet and triplet states (Fig. S14†) and then represented as a function of the reaction coordinate *α* as shown in Fig. 6A.

Reactants (1) are stable in the triplet state arising from the combination of the electronic ground state of O_2_(^3^Σ_g_^−^), and the ground state of QND (singlet, all electrons are paired). However, as QND and O_2_ move closer to each other, the energy of the system increases and any attempt to optimize C2-peroxide in the triplet state led back to the reactants. In contrast, formation of the peroxide in the singlet state proceeds barrierless leading to a stable moiety. Analysis of the singly occupied molecular orbitals (Fig. S15†) suggests that stabilization and destabilization of the singlet state and of the triplet state, respectively, originate from the combination of one π* orbital of O_2_ with the π cloud of QND. This leads to an orbital with antibonding character along the C2–O2 and O1–O2 bonds, which is singly occupied in the triplet state and unoccupied in the singlet state. Singlet and triplet states must cross at some point along the reaction path and the minimum energy crossing point (MECP, highlighted by a black dot in Fig. 6A) acts as the effective transition state. Our calculations locate the MECP (2) at 10.2 kcal mol^−1^ above the energy of the activated QND–O_2_ complex (1). At the MECP, *d*(C2–O2) is 1.62 Å, and QND is no longer planar. To get further insight into the ISC stage, we evaluated how *δ*(O2–C2) and *δ*(O1–O2) change along the reaction path in both the singlet and triplet states (left-hand panel in Fig. 6B). We observed that shortening of *d*(C2–O2), results in an increase in the electronic density shared between the substrate and dioxygen, which leads to the weakening of the O_2_ bond, a consequence of the larger charge transfer from QND to O_2_. Moreover, at every point along the reaction path, charge transfer is stronger in the singlet than in the triplet state, so the spin-flip involves a significant transfer of electronic density between QND and O_2_.

Once the peroxide is formed, the next stage of the reaction proceeds *via* the formation of endoperoxide (3) and subsequent rupture of the C2–C3 and C3–C4 bonds, followed by CO release that is very exothermic. The process is again well described by the delocalization indices (right-hand panel in Fig. 6B). Once the system is in the singlet state, the peroxide attacks the carbonyl group establishing the C4–O1 bond of endoperoxide. At the barrier (4), concerted breaking of endoperoxide and CO release takes place. As shown in Fig. 6B, the energy of the transition state for the CO-release stage lies 10.8 kcal mol^−1^ above the reactants, which is slightly above the energy of the MECP. However, to compare the barrier heights it is necessary to correct the ISC energy barrier for the probability of hopping from the triplet to the singlet state,^34^ which depends on the value of the spin–orbit coupling (SOC). At the MECP, we calculated a SOC of 22 cm^−1^, leading to a swapping probability of 0.005, *i.e.* an increase of 3.1 kcal mol^−1^ of the barrier (see the ESI† for further details). Therefore, the ISC stage is the rate limiting step of the overall process.

We also considered the possibility of a direct electron transfer within the complex by calculating the energy difference between [^3^O_2_ + ^1^QND^−^] and [^2^O_2_˙ + ^2^QND˙] at the reactants' asymptote, based on separate calculations for the donor and the acceptor. We obtained a value of 17 kcal mol^−1^, which is significantly larger than the energy of the MECP (even when corrected by the swapping probability). This result is compatible with our findings that, along the reaction path, the energy of the singlet state monotonically decreases. Although such an endothermicity does not allow the possibility of a direct electron transfer to be completely ruled out,^19^ our calculations predict a scenario where spin-flipping is more favorable. Consistent with this, no significant amounts of radical species were detected in spin-trapping experiments with wild-type HOD.^35^

## Concluding remarks

The “great oxidation event” 2.4 billion years ago led to the permanent accumulation of O_2_ in the atmosphere, thus exerting evolutionary pressure on enzyme systems that could take advantage of such a strong oxidant. A very recent KEGG (Kyoto Encyclopedia of Genes and Genomes) analysis of the 136 Pfam families of all known O_2_-dependent enzymes, found that only ∼60% of these can be classified as having a function primarily related to O_2_ whilst the remaining ∼40% represent families featuring only sporadic O_2_ utilizers.^2^ The latter group has therefore likely evolved O_2_-metabolizing capabilities from a different set of original catalytic competences. Hydrolases, particularly metallo-β-lactamases and those of the ABH-fold, appear to be the most common progenitors.^2^ ABH-fold dioxygenases are particularly intriguing as they accomplish O_2_ redox chemistry without external cofactors.

Here, using HOD as a paradigm for the growing family of ABH-fold dioxygenases, we have shown how evolution has taken advantage of multiple structural elements of the ABH-fold to enable the switch from hydrolytic to oxygenolytic reactions. A key requirement for such catalytic repositioning is the ability to host O_2_ sufficiently close to its *N*-heteroaromatic substrate for the reaction to take place. Using *in crystallo* O_2_-pressure freeze-trapping we have visualized the ternary complex with a substrate analog at high resolution, thus providing conclusive evidence that the oxyanion hole fulfills the role of an O_2_ receptor. Computational analyses of dioxygen diffusion pathways in various non-ABH-fold enzyme systems including flavoenzymes,^36^ the nonheme iron-containing 12/15-lipoxygenase,^37^ and also the metal-independent DpgC dioxygenase^21^ have uncovered some common themes in O_2_ access strategies, often involving a network of transient pathways that are mostly lined with hydrophobic residues. In HOD, we have identified a set of pockets that provide a route for O_2_ access to the oxyanion hole in the E–S complex. However, compared to other systems, O_2_ entry appears a much rarer event arguing in favor of additional strategies to increase the local O_2_ concentration. In urate oxidase, a hydrophobic cavity adjacent to the active site has been suggested to act as a transient O_2_ reservoir.^38^ We propose that in HOD, and likely in all ABH-fold dioxygenases that operate on similar organic substrates, the active site itself acts as a temporary O_2_ binding site. Transient O_2_ storage at the xenon-validated B-site that we have identified overlapping with the hydrophobic portion of the *N*-heteroaromatic binding site represents a convenient solution to the problem of O_2_ localization, as one might envisage a mechanism of displacement that upon substrate binding transfers O_2_ from the B-site to the R-site that is only about 5 Å away, thus organizing both reactants for catalysis.

ABH-fold enzymes often employ their typical Ser-His-Asp triad to catalyze reactions following an esterase-type mechanism. Departures from this have however been observed in some non-hydrolytic reactions. *Manihot esculenta* (cassava) and *Hevea brasiliensis* (rubber tree) hydroxynitrile lyases that break the C–C bond in cyanohydrin although featuring the classical Ser-His-Asp triad, they use their active site serine as a general base. Even more dramatic is tomato methyl ketone (MK) synthase (MKS1) involved in MK 2-tridecanone synthesis that lacks the canonical triad whilst retaining a conserved histidine residue that acts as the catalytic base with a neighboring threonine providing the H-bond bond pattern required for the decarboxylation step of β-keto acid substrates.^39^ For oxygenation, the ABH-fold catalytic machinery is also employed in an unconventional manner and substrates' activation by deprotonation of their 3-hydroxyl group only requires the His-Asp subset (H251-D126 in HOD) that acts as a general base.^4,16^ Although HOD's serine residue (S101) has been shown to retain nucleophilic properties in some hydrolase-like reactions,^40^ this capability is irrelevant for oxygenation.^4^ Instead, our results indicate that in addition to a contribution in the stabilization of the organic reactant,^4^ S101 plays a role as an O_2_/H_2_O modulator with its *trans* rotamer promoting both O_2_ stabilization and H_2_O destabilization in the oxyanion hole. Substitution of the cryptic serine nucleophile with an alanine, a replacement naturally present in some ABH-fold dioxygenases that further strengthens the argument of nucleophile dispensability, also promotes O_2_ stabilization at the catalytic site. In HOD, S101A replacement, however, has a negative impact on the *K*_M_ value of its organic substrate (60-fold increase compared to that of wild-type HOD) whilst *k*_cat_ is not affected.^4^ This suggests that the evolutionary choice of the specific amino acid at the ‘nucleophile elbow’ is likely the result of a combination of factors aimed at maximizing the stability of the organic substrate and O_2_ whilst achieving destabilization of H_2_O.

Deprotonation of the substrate is a common step in cofactor-independent oxygenation^14,41^ and our QM and QM/MM analyses reveal that the charge of the substrate determines the geometry of the O_2_–substrate complex. Cofactorless addition of O_2_ to QND requires swapping from the triplet to the singlet state, a “spin-forbidden” process. The degree of spin-forbiddenness is determined by the magnitude of the SOC term which correlates with the energy difference between the two π-orbitals of O_2_. When QND is deprotonated, the geometry of the O_2_–QND complex is that which maximizes electron density transfer from QND (π) to O2 (π*) orbitals, thus perturbing the π-symmetry of O_2_ and leading to values of SOC large enough to make the reaction feasible,^42^ even in the absence of any metal cofactor. For spin-forbidden reactions, the MECP acts as the main dynamical bottleneck for the reaction,^34^ and we calculated that the additional burden caused by the spin-forbidden character of the reaction is 3.1 kcal mol^−1^. Similarly to what was obtained for DpgC,^43^ vitamin K-dependent glutamate carboxylase,^44^ or nogalamycin monoxygenase,^45^ O_2_ is not protonated at the MECP, in contrast to what was obtained for *p*-hydroxyphenylacetate hydroxylase, a flavin-dependent monooxygenase.^46^

To summarize, evolution has repurposed the ABH-fold architecture and its simple catalytic machinery to accomplish metal-independent oxygenation. This is achieved by dioxygen entrapment at the oxyanion hole in proximity to the organic ligand with its stabilization and concomitant H_2_O destabilization modulated by the nucleophile/hydrophobic elbow residue (Ser/Ala). Substrate deprotonation mediated by the His-Asp subset of the catalytic triad elicits a ligand-O_2_ geometry that maximizes electron transfer such that the spin-forbiddenness of the direct reaction is relaxed.

## Data availability

Crystallographic structures have been deposited with the Protein Data Bank with accession codes 7OJM, 7OKZ, 8A97, 8ORO, 8OXN, 8OXT.

## Author contributions

RAS conceived the project. SB performed the experimental work. SGG, MC, and PGJ carried out the computational work. PvdL and PC contributed to the crystal pressurization experiments. SB, SGG, MC, PGJ, and RAS analyzed the data. The manuscript was written by RAS with contributions from MC and PGJ and commented on by all authors.

## Conflicts of interest

The authors declare no competing interests.

## Supplementary Material

SC-014-D3SC03044J-s001

## References

[cit1] Iyer L. M., Abhiman S., de Souza R. F., Aravind L. (2010). Origin and evolution of peptide-modifying dioxygenases and identification of the wybutosine hydroxylase/hydroperoxidase. Nucleic Acids Res..

[cit2] Jablonska J., Tawfik D. S. (2022). Innovation and tinkering in the evolution of oxidases. Protein Sci..

[cit3] Sattler S. A., Wang X., Lewis K. M., DeHan P. J., Park C. M., Xin Y., Liu H., Xian M., Xun L., Kang C. (2015). Characterizations of two bacterial persulfide dioxygenases of the metallo-beta-lactamase superfamily. J. Biol. Chem..

[cit4] Steiner R. A., Janssen H. J., Roversi P., Oakley A. J., Fetzner S. (2010). Structural basis for cofactor-independent dioxygenation of *N*-heteroaromatic compounds at the alpha/beta-hydrolase fold. Proc. Natl. Acad. Sci. U. S. A..

[cit5] Lenfant N., Hotelier T., Velluet E., Bourne Y., Marchot P., Chatonnet A. (2013). ESTHER, the database of the alpha/beta-hydrolase fold superfamily of proteins: tools to explore diversity of functions. Nucleic Acids Res..

[cit6] Heikinheimo P., Goldman A., Jeffries C., Ollis D. L. (1999). Of barn owls and bankers: a lush variety of alpha/beta hydrolases. Structure.

[cit7] Nardini M., Dijkstra B. W. (1999). Alpha/beta hydrolase fold enzymes: the family keeps growing. Curr. Opin. Struct. Biol..

[cit8] Bugg T. D. (2004). Diverse catalytic activities in the alphabeta-hydrolase family of enzymes: activation of H_2_O, HCN, H_2_O_2_, and O_2_. Bioorg. Chem..

[cit9] Bauer I., Max N., Fetzner S., Lingens F. (1996). 2,4-dioxygenases catalyzing *N*-heterocyclic-ring cleavage and formation of carbon monoxide. Purification and some properties of 1*H*-3-hydroxy-4-oxoquinaldine 2,4-dioxygenase from Arthrobacter sp. Ru61a and comparison with 1*H*-3-hydroxy-4-oxoquinoline 2,4-dioxygenase from *Pseudomonas putida* 33/1. Eur. J. Biochem..

[cit10] Fischer F., Kunne S., Fetzner S. (1999). Bacterial 2,4-dioxygenases: new members of the alpha/beta hydrolase-fold superfamily of enzymes functionally related to serine hydrolases. J. Bacteriol..

[cit11] Birmes F. S., Wolf T., Kohl T. A., Ruger K., Bange F., Kalinowski J., Fetzner S. (2017). *Mycobacterium abscessus* subsp. abscessus is capable of degrading *Pseudomonas aeruginosa* quinolone signals. Front. Microbiol..

[cit12] Muller C., Birmes F. S., Ruckert C., Kalinowski J., Fetzner S. (2015). *Rhodococcus erythropolis* BG43 genes mediating pseudomonas aeruginosa quinolone signal degradation and virulence factor attenuation. Appl. Environ. Microbiol..

[cit13] Wullich S. C., Arranz San Martin A., Fetzner S. (2020). An alpha/beta-hydrolase fold subfamily comprising pseudomonas quinolone signal-cleaving dioxygenases. Appl. Environ. Microbiol..

[cit14] Fetzner S., Steiner R. A. (2010). Cofactor-independent oxidases and oxygenases. Appl. Microbiol. Biotechnol..

[cit15] Frerichs-Deeken U., Ranguelova K., Kappl R., Huttermann J., Fetzner S. (2004). Dioxygenases without requirement for cofactors and their chemical model reaction: compulsory order ternary complex mechanism of 1*H*-3-hydroxy-4-oxoquinaldine 2,4-dioxygenase involving general base catalysis by histidine 251 and single-electron oxidation of the substrate dianion. Biochemistry.

[cit16] Hernandez-Ortega A., Quesne M. G., Bui S., Heuts D. P., Steiner R. A., Heyes D. J., de Visser S. P., Scrutton N. S. (2014). Origin of the proton-transfer step in the cofactor-free (1*H*)-3-hydroxy-4-oxoquinaldine 2,4-dioxygenase: effect of the basicity of an active site His residue. J. Biol. Chem..

[cit17] Wullich S. C., Kobus S., Wienhold M., Hennecke U., Smits S. H. J., Fetzner S. (2019). Structural basis for recognition and ring-cleavage of the *Pseudomonas quinolone* signal (PQS) by AqdC, a mycobacterial dioxygenase of the alpha/beta-hydrolase fold family. J. Struct. Biol..

[cit18] Hernandez-Ortega A., Quesne M. G., Bui S., Heyes D. J., Steiner R. A., Scrutton N. S., de Visser S. P. (2015). Catalytic mechanism of cofactor-free dioxygenases and how they circumvent spin-forbidden oxygenation of their substrates. J. Am. Chem. Soc..

[cit19] Silva P. J. (2016). Refining the reaction mechanism of O_2_ towards its co-substrate in cofactor-free dioxygenases. PeerJ.

[cit20] Dimitriou P. S., Denesyuk A. I., Nakayama T., Johnson M. S., Denessiouk K. (2019). Distinctive structural motifs co-ordinate the catalytic nucleophile and the residues of the oxyanion hole in the alpha/beta-hydrolase fold enzymes. Protein Sci..

[cit21] Di Russo N. V., Condurso H. L., Li K., Bruner S. D., Roitberg A. E. (2015). Oxygen diffusion pathways in a cofactor-independent dioxygenase. Chem. Sci..

[cit22] Johnson B. J., Cohen J., Welford R. W., Pearson A. R., Schulten K., Klinman J. P., Wilmot C. M. (2007). Exploring molecular oxygen pathways in *Hansenula polymorpha* copper-containing amine oxidase. J. Biol. Chem..

[cit23] Kallio J. P., Rouvinen J., Kruus K., Hakulinen N. (2011). Probing the dioxygen route in *Melanocarpus albomyces* laccase with pressurized xenon gas. Biochemistry.

[cit24] Chaloupkova R., Liskova V., Toul M., Markova K., Sebestova E., Hernychova L., Marek M., Pinto G. P., Pluskal D., Waterman J., Prokop Z., Damborsky J. (2019). Light-emitting dehalogenases: reconstruction of multifunctional biocatalysts. ACS Catal..

[cit25] Colloc'h N., Gabison L., Monard G., Altarsha M., Chiadmi M., Marassio G., Sopkova-de Oliveira Santos J., El Hajji M., Castro B., Abraini J. H., Prange T. (2008). Oxygen pressurized X-ray crystallography: probing the dioxygen binding site in cofactorless urate oxidase and implications for its catalytic mechanism. Biophys. J..

[cit26] Roeser D., Preusser-Kunze A., Schmidt B., Gasow K., Wittmann J. G., Dierks T., von Figura K., Rudolph M. G. (2006). A general binding mechanism for all human sulfatases by the formylglycine-generating enzyme. Proc. Natl. Acad. Sci. U. S. A..

[cit27] Roeser D., Schmidt B., Preusser-Kunze A., Rudolph M. G. (2007). Probing the oxygen-binding site of the human formylglycine-generating enzyme using halide ions. Acta Crystallogr., D: Biol. Crystallogr..

[cit28] Lovell S. C., Word J. M., Richardson J. S., Richardson D. C. (2000). The penultimate rotamer library. Proteins.

[cit29] Lafumat B., Mueller-Dieckmann C., Leonard G., Colloc’h N., Prangé T., Giraud T., Dobias F., Royant A., van der Linden P., Carpentier P. (2016). Gas-sensitive biological crystals processed in pressurized oxygen and krypton atmospheres: deciphering gas channels in proteins using a novel `soak-and-freeze' methodology. J. Appl. Cryst..

[cit30] Bui S., von Stetten D., Jambrina P. G., Prange T., Colloc'h N., de Sanctis D., Royant A., Rosta E., Steiner R. A. (2014). Direct evidence for a peroxide intermediate and a reactive enzyme-substrate-dioxygen configuration in a cofactor-free oxidase. Angew Chem., Int. Ed. Engl..

[cit31] Khaliullin R. Z., Cobar E. A., Lochan R. C., Bell A. T., Head-Gordon M. (2007). Unravelling the origin of intermolecular interactions using absolutely localized molecular orbitals. J. Phys. Chem. A.

[cit32] Horn P. R., Head-Gordon M. (2015). Polarization contributions to intermolecular interactions revisited with fragment electric-field response functions. J. Chem. Phys..

[cit33] Gil-Guerrero S., Melle-Franco M., Peña-Gallego Á., Mandado M. (2020). Clar goblet and aromaticity driven multiradical nanographenes. Chem.–Eur. J.

[cit34] Harvey J. N. (2007). Understanding the kinetics of spin-forbidden chemical reactions. Phys. Chem. Chem. Phys..

[cit35] Thierbach S., Bui N., Zapp J., Chhabra S. R., Kappl R., Fetzner S. (2014). Substrate-assisted O2 activation in a cofactor-independent dioxygenase. Chem. Biol..

[cit36] Baron R., Riley C., Chenprakhon P., Thotsaporn K., Winter R. T., Alfieri A., Forneris F., van Berkel W. J., Chaiyen P., Fraaije M. W., Mattevi A., McCammon J. A. (2009). Multiple pathways guide oxygen diffusion into flavoenzyme active sites. Proc. Natl. Acad. Sci. U. S. A..

[cit37] Saam J., Ivanov I., Walther M., Holzhutter H. G., Kuhn H. (2007). Molecular dioxygen enters the active site of 12/15-lipoxygenase *via* dynamic oxygen access channels. Proc. Natl. Acad. Sci. U. S. A..

[cit38] Colloc'h N., Prange T. (2014). Functional relevance of the internal hydrophobic cavity of urate oxidase. FEBS Lett..

[cit39] Auldridge M. E., Guo Y., Austin M. B., Ramsey J., Fridman E., Pichersky E., Noel J. P. (2012). Emergent decarboxylase activity and attenuation of alpha/beta-hydrolase activity during the evolution of methylketone biosynthesis in tomato. Plant Cell.

[cit40] Thierbach S., Buldt-Karentzopoulos K., Dreiling A., Hennecke U., Konig S., Fetzner S. (2012). Hydrolase-like properties of a cofactor-independent dioxygenase. Chembiochem.

[cit41] Bui S., Steiner R. A. (2016). New insight into cofactor-free oxygenation from combined experimental and computational approaches. Curr. Opin. Struct. Biol..

[cit42] Thorning F., Jensen F., Ogilby P. R. (2022). Geometry dependence of spin–orbit coupling in complexes of molecular oxygen with atoms, H2, or organic molecules. J. Phys. Chem. A.

[cit43] Ortega P., Zanchet A., Sanz-Sanz C., Gomez-Carrasco S., Gonzalez-Sanchez L., Jambrina P. G. (2021). DpgC-catalyzed peroxidation of 3,5-dihydroxyphenylacetyl-CoA (DPA-CoA): insights into the spin-forbidden transition and charge transfer mechanisms. Chem.–Eur. J..

[cit44] Silva P. J., Ramos M. J. (2007). Reaction mechanism of the vitamin K-dependent glutamate carboxylase: a computational study. J. Phys. Chem. B.

[cit45] Cantu Reinhard F. G., DuBois J. L., de Visser S. P. (2018). Catalytic mechanism of nogalamycin monoxygenase: how does nature synthesize antibiotics without a metal cofactor?. J. Phys. Chem. B.

[cit46] Visitsatthawong S., Chenprakhon P., Chaiyen P., Surawatanawong P. (2015). Mechanism of oxygen activation in a flavin-dependent monooxygenase: a nearly barrierless formation of C4a-hydroperoxyflavin *via* proton-coupled electron transfer. J. Am. Chem. Soc..

