# Supplementary material for: Evolutionary adaptation from hydrolytic to oxygenolytic catalysis at the α/β-hydrolase fold
Source: Chem Sci. 2023 Sep 18;14(38):10547–60. doi: 10.1039/d3sc03044j (PMC10548524; doi:10.1039/d3sc03044j)
Supplement: SC-014-D3SC03044J-s001 [file SC-014-D3SC03044J-s001.pdf]

Electronic Supplementary Material (ESI) for Chemical Science.
This journal is © The Royal Society of Chemistry 2023

Supplementary Information for

Evolutionary adaptation from hydrolytic to oxygenolytic catalysis at the α/β-hydrolase fold

Soi Bui[a], Sara Gil-Guerrero[b], Peter van der Linden[c], Philippe Carpentier[d,e], Matteo Ceccarelli[f,g], Pablo G. Jambrina[b], Roberto A. Steiner[a,h]

[a]Randall Centre for Cell and Molecular Biophysics, King's College London, London, SE1 1UL, UK

[b]Departamento de Química Física, University of Salamanca, Salamanca, 37008, Spain

[c]European Synchrotron Radiation Facility (ESRF), Partnership for Soft Condensed Matter (PSCM), 71 Avenue des Martyrs, Grenoble 38043, France

[d]European Synchrotron Radiation Facility (ESRF), 71 Avenue des Martyrs, 38043 Grenoble, France

[e]Université Grenoble Alpes, CNRS, CEA, Interdisciplinary Research Institute of Grenoble (IRIG), Laboratoire Chimie et Biologie des Métaux (LCBM) UMR 5249, 17 Avenue des Martyrs, 38054 Grenoble, France

[f]Department of Physics, University of Cagliari, 09042 Monserrato, Italy

[g]IOM-CNR Unità di Cagliari, Cittadella Universitaria, 09042 Monserrato, Italy

[h]Department of Biomedical Sciences, University of Padova, Italy

Corresponding authors

Matteo Ceccarelli matteo.ceccarelli@dsf.unica.it

Pablo G. Jambrina pjambrina@usal.es

Roberto A. Steiner roberto.steiner@kcl.ac.uk or roberto.steiner@unipd.it

DNA techniques, protein expression, and protein purification

For protein overexpression in *E. coli*, the target gene was inserted into the pQE30 vector (Qiagen) at the BamHI and SalI restriction sites. An uncleavable N-terminal His_6 fusion tag of sequence MRGSHHHHHHGS was added to the gene product to facilitate protein purification. As wild-type HOD tends to dimerize due to oxidative formation of an intermolecular disulfide bridge, we took advantage of the C69S substitution that abrogates this problem.[1] The HOD C69S variant (HOD^{C69S}) has catalytic properties that are identical to those of wild-type HOD. All work described here was carried out in this background. For simplicity, throughout the text, we refer to His_6-HOD^{C69S} simply as HOD. Site-directed mutagenesis to generate all HOD variants was performed using standard techniques followed by gene sequencing for validation. Protein over-expression was accomplished in *E. coli* M15[pREP4] cells at 28 °C. Purification of all HOD variants was performed using a combination of chromatographic techniques as described previously. The substrate 1-*H*-3-hydroxy-4-oxoquinaldine (QND) was a kind gift of Professor S. Fetzner (University of Münster, Germany). The substrate analogue 2-methyl-quinolin-4(1*H*)-one (MQO) was commercially synthesized by AF ChemPharm, Sheffield, UK.

X-ray crystallography

Crystals of HOD, HOD^{H251A}, HOD^{S101A} exhibiting variable degree of twinning and belonging to space group $P2_12_12_1$ or $P4_1$ were obtained by the vapor-diffusion method at 18 °C by mixing equal volumes of protein solution (150 mg/ml in 20 mM Tris-HCl,100 mM NaCl, 2 mM EDTA, 1mM DTT, pH 7.5) and a reservoir solution containing 1.65 M Na/K tartrate and 100 mM Hepes, pH 7.0. Complexes with the organic compounds were obtained by soaking crystals in a reservoir solution enriched by a 2 mM solution of the appropriate ligand for typically around six hours. Noble gas derivatization was achieved by pressurizing at 30 bar a capillary-mounted HOD crystal in pure Xe atmosphere. Pressure was maintained for the entire duration

of the room-temperature diffraction experiment. For dioxygen pressurization experiments, we employed the methodology dubbed 'soak-and-freeze'.[2] Crystals were mounted in specific sample high pressure-supports (SPINE standard pins with a capillary) and exposed to 40 bar O_2 for two minutes at ambient temperature prior to cryo-cooling in liquid N_2 whilst still under pressure. Except for the Xe pressurization measurement, all data collections were performed at 100K with crystals cryo-protected using the reservoir condition spiked with 20% glycerol (v/v). Complete data sets were measured at beam lines I02, I04-1, I24 of Diamond Light Source (Didcot, United Kingdom) or ID29, BM30A of the European Synchrotron Radiation Facility (Grenoble, France). Data processing was carried out using the *xia2* pipeline[3] and crystal structures solved either by the molecular replacement technique using *Phaser*[4] or by difference Fourier methods. Model refinement was achieved with the *Refmac*5[5] package. Model building was performed with COOT.[6] A summary of data collection and refinement statistics are shown in Table S1.

In crystallo turnover

In solution, the H251A replacement renders the enzyme almost inactive (k_{cat} of 0.0006 s^{-1} at pH 6.5 compared to 16.3 s^{-1} for wtHOD) as it lacks the functional H251-D126 dyad required for the fast substrate activation step in which its 3OH group is deprotonated.[7, 8] Previously, we have obtained the X-ray structure of the HOD^{H251A}-QND complex under normoxic conditions.[7] The structure shows that under these conditions the substrate binds in the active site without any appreciable turnover. Despite the very limited activity of HOD^{H251A}, O_2 pressurization of HOD^{H251A}-QND crystals (40 bar for 2 minutes) results in complete turnover and electron density maps at 2.0 Å show the bound N-acetylanthranilate product (Figure S8A). This experiment clearly demonstrates that O_2 can reach the *R*-site in the preformed E-S complex promoting QND conversion into the NAA product during pressurization. Reactivity must

therefore be attributed to a small quantity of the QND anion present at the equilibrium. *In crystallo* UV/Vis spectra are consistent product formation without substantial accumulation of the substrate anion (Figure S8B). The structure suggests that proton abstraction is mediated by solvent molecules that connect the 3OH group of the substrate with D126 (Figure S8A). UV/Vis spectra shown in (Figure S8B) were recorded using the *in crystallo* spectroscopic facility at ESRF.[9]

Standard molecular dynamics (MD) simulations

Initial coordinates for substrate-free simulations were taken from the relevant crystallographic structure (PDB code 2WJ3). Hydrogen atoms were added by the antechamber module of the AMBER package using geometric rules.[10] The protonation state of histidine residues and other amino acids were checked using the PROPKA tool.[11, 12] The protonation state of residue H251 was chosen in relation to the charge of the substrate. The position of hydrogens on the histidine ring (delta/epsilon) were determined by visual inspection of the high-resolution structure looking at possible hydrogen bonds with nearest residues (epsilon: 7, 40, 100, 102, 154, 164, 171, 238, 264; delta: 38, 64, 172, 217, 251 when neutral). The enzyme was solvated by in a cubic box of 85×85×85 Å^3, with water molecules placed initially 5 Å away from the protein surface whilst retaining all crystallographic water molecules. Fifteen sodium ions were added to neutralize the protein charge. The system was initially relaxed using the NAMD package[13] while production runs were performed with ACEMD.[14] For the first 2 ns, restraints were applied to both Cα and Cβ protein carbon atoms and to crystallographic waters. At T = 300 K in the NPT ensemble, all restraints were gradually removed over 4 ns. After 2 ns in the NPT ensemble at 300 K and P = 1 atm, we calculated the average size of the box and we used this value for simulating the system in the NVT ensemble (L=73.67 Å, 39081 atoms). Long-range electrostatic interactions were treated with the Soft-Particle-Mesh-Ewald algorithm[15] (64 grid

points with direct cutoff at 9.0 Å, as suggested by the ACEMD software; the same cutoff holds for van der Waals interactions). We employed the Amber99SB ILDN forcefield[16] and TIP3P[17] for the protein and water molecules, respectively. The GAFF force-field parameters[18] were used to describe the organic molecules. Partial atomic charges were evaluated according to the RESP approach:[19] the molecule was first optimized at the HF/6-31G(d) level, up to a convergence in energy of 10^{-5} au, using the Gaussian software.[20] Atomic RESP charges were derived from the electrostatic potential using the antechamber module of the AMBER package.[10] O_2 was parametrized with the LJ Amber parametrization, by adding a small charge to have a dipole of 0.121 eÅ.

Two independent 1 μs-long replica simulations were carried out without or with explicit O_2 (WAT-MD and OXY-MD, respectively). For OXY-MD, ten O_2 molecules were added to the system in the cubic box with L = 73.67 Å. Normalized dioxygen distribution was calculated by sampling O_2 positions every 50 ps onto a matrix of $0.6 \times 0.6 \times 0.6$ Å^3 elementary cubes and dividing the total atom count in each volume by the total number of frames.

Initial coordinates for substrate-bound simulations were taken from the anaerobic HOD-QND complex (PDB code 2WJ4) and the system was setup as described above. QND was initially considered in its protonated form at O3 (QNDH) with neutral H251 protonated on ND1. The force field for the substrate was obtained as described previously.[21] The protonation of all other histidine residues was chosen to preserve the H-bond pattern observed in the X-ray structure. At 300 K in the NPT ensemble, we removed protein restraints over 4 ns and transferred the proton from the substrate's O3 atom to H251(NE2) as this represents the mechanistically-relevant complex.[7] Weak restraints on the substrate positioning were maintained throughout to prevent its escape from the active site during the runs. To study O_2 entry in the HOD-QND complex we performed a total of 80 independent 200-ns MD runs by adding to the system either ten O_2 molecules in the L = 73.67 Å box (OXY10-S-MD runs) or

one O_2 molecule confined in a sphere with a 17-Å radius of centered on S101 (OXY1-S-MD runs). Of the 40 OXY10-S-MD runs, 25 were carried out with a harmonic restrains on S101 to maintain its *trans* rotamer and 15 had no restraints on S101. Of the 40 OXY1-S-MD runs, 20 were carried out with a harmonic restrains on S101 to maintain its *trans* rotamer and 20 had no restraints on S101.

Metadynamics Simulations

To investigate the oxygen diffusion within HOD, we also performed metadynamics simulations employing a bias on the O_2 position.[22] As coordinates to be biased we selected the position of the dioxygen center of mass in the 3D Cartesian space. As consequence of the use of absolute coordinates, we kept the protein frame in place by restraining the position of a set of Cα atoms (residues 31, 101, 112, 243) to the reference structure obtained after relaxation. We performed well-tempered metadynamics,[23] DT= 3000 K, with bias added as Gaussians with a frequency of 1 ps. As the *R*-site cavity is rather small, we decided to work with narrow Gaussians (width=0.3 Å). For this reason, we started with a high value for the initial height (1 kcal/mol) and this value is smoothly decreased by the well-tempered algorithm during long runs. Dioxygen was confined to a sphere of 17-Å radius centered on S101. We performed three simulations of 1.8 μs each. In two replica runs (identical conditions except for initial velocities chosen randomly) S101 was restrained to its *trans* rotamer. No restraints were employed in the third one. We calculated the error on metadynamics by reweighting the positions sampled during the runs with the corresponding bias term and comparing the free energy surface with the one obtained integrating the bias potential.

Analysis of O_2 and H_2O escape times from the *R*-site

Preliminary tests indicated that O_2 residence times at the *R*-site are in the order of ns, corresponding to an energy barrier of few kcal/mol, achievable with thermal motions (kT = 0.6 kcal/mol at 300 K). Thus, the use of enhanced sampling techniques such as metadynamics[24] or TPS-derived methods[25] was not required for this analysis. As our starting point we took advantage of a protein state found in our OXY1-S-MD run in which O_2 was found at the *R*-site. This was subjected to 200 steps of conjugate gradient minimization and then thermalised for 1 ns at 300 K before assigning new random velocities to 50 independent standard MD simulations. For each trajectory we evaluated the time spent by O_2 at the *R*-site before it abandons this location. The escape process can be seen as a diffusion process over a barrier dividing two basins. Although there are uncertainties in the estimation of the precise time when a molecule goes over the unknown transition state for calculating the residence time,[25] we can associate to the transition over a barrier the abrupt change in a collective variable/parameter. By plotting the distance of oxygen from the substrate geometric center (calculated using heavy atoms only), we recognized that around 6 Å there is an abrupt change of position. Thus, we defined the residence time as the time to reach in the sampled trajectories that distance, without considering the possibility to recross back the transition state or considering the transmission coefficient equal to one. Harmonic restraints were employed to preserve S101 rotamers and the substrate position (on average) during the simulations.

Escape times (k_{off}^{-1}) are expected to follow the exponential distribution:

$$f(t) = \begin{cases} \lambda e^{-\lambda t}; t > 0 \\ 0; t \leq 0 \end{cases}; < t >= \frac{1}{\lambda}; \sqrt{\sigma^2(t)} = \frac{1}{\lambda}$$

belonging to the family of Poisson statistics. From this distribution we can derive the probability that an event occurs before time t:

$$F(t) = P(T \leq t) = \int_0^t \lambda e^{-\lambda T} dT = 1 - e^{-\lambda t}.$$

Numerically we can fit F(t) with the cumulative histogram of escape times to obtain the single parameter lambda that characterizes this distribution. We can also determine the average escape time using block averages. Fitting the cumulative distribution is however more appropriate because it is difficult to sample long escape times. The cumulative distributions were obtained by accumulating data with a bin width of 1 ns.

QM calculations of the isolated ligand-O_2 complexes

DFT calculations to determine the equilibrium geometries of the ligand-O_2 complexes were carried out at the M062X/6-31+G(d,p) level of theory using QChem 5.2[26] using the D3(0) dispersion correction.[27] Since even for simple systems, such as toluene-O_2 there are many conformers possible,[28] we have reduced the dimensionality of the problem by restraining the sum of the (C2-O1) and (C4-O2) ligand-O_2 distances to their crystallographic values. We find however that around the equilibrium geometry, results are quite insensitive to small changes in the restrain value (Figure S10). This restraint was not applied in the QM/MM calculations, as the protein scaffold prevents O_2 motion away from its pocket. Energy decomposition analysis (EDA)[29] as implemented in QChem 5.2 was employed to calculate the interaction energy between O_2 and QND/MQO, and its contributing terms. Delocalization indices[30] (δ) using the partitioning Mulliken scheme[31] were calculated using the NDELOC code,[32] interfaced with Gaussian16.[20]. They are a measure of 'electron sharing' between sets of atomic centers, and their 2-order magnitudes allowed us to assign "bond orders". To avoid a wrong partition within the Hilbert space, diffuse functions were not included in δ values calculations.

QM/MM energy minimizations

The HOD-MQO/QND-O_2 ternary complexes used as starting point for the QM/MM calculations were prepared from the relevant crystallographic structures using the CHARMM

software[33] and the CHARMM36 forcefield.[34] MD simulations were performed with NAMD 2.13.[13] The coordinates of the hydrogen atoms were generated using the standard protonation states for the titrable residues. H251 was protonated in the HOD-QND system, as this residue abstracts the 3OH proton from QND. The protein complexes were placed in the center of a cubic TIP3 water box, large enough to include the protein and at least 10 Å of solvent on all sides. KCl at a concentration of 0.15 M was added to neutralize the system, and the Particle Mesh Ewald method[35] was used to treat the electrostatics of the periodic boundary conditions. The system was subjected to a classical MD equilibration of 10 ns at constant temperature (303.15 K) and pressure (1 atm), where to avoid O_2 diffusion outside the active site, the position of QND/MQO and O_2 were restrained throughout the equilibration. After equilibration, the system was trimmed to a sphere of 26 Å centered around O_2, where the atoms further away than 18 Å were kept frozen during all QM/MM calculations. Full electrostatic embedding was adopted, and hydrogen link atoms were used to treat the QM/MM boundaries, when necessary. QM calculations were carried out at the M062X/6-31+G(d,p) level of theory using Grimme's dispersion correction as in the gas-phase calculations. In a first set of calculations, the reactants were optimized and, subsequently, the (C4-C2-O2-O1) dihedral angle (Φ) was scanned through geometry restrained minimizations. For this study, the QM region included the substrate, O_2, the sidechain of H251 (protonated for QND-O_2 simulation), H102, G103, the backbone of G35, W36, C37, and three water molecules close to either O_2 or the histidine sidechain. No restraints were applied the (C2-O1) and (C4-O2) distances. In a second set of calculations, we calculated the energy profile along the reaction path. For the CO-release stage, we used as a reaction coordinate α = d(O1-O2) + d(C3-C4) + d(C2-C3) – d(O1-C4) – d(O2-C2), which includes all bond distances that are formed and broken during the reaction. Calculations with and without the additional restraint of d(C3-C4) = d(C2-C3) were run, showing that including this restraint aids to reach the product state, without modifying the barrier heights. To ensure convergence,

geometry restrained minimizations were carried out forwards and backwards. Calculations were carried out using two different QM regions, one including just the substrate (QND or MQO) and O_2, and another also including the sidechain of H100, S101, and H251 as well as a water molecule nearby. The results were found to be relative insensitive to the size of the QM region. To describe the intersystem crossing (ISC) stage, the energy difference between the singlet and triplet states was chosen as a reaction coordinate,[36] so the energy of the Minimum Energy Crossing Point (MECP) is that obtained when the energy difference is zero. The results are shown in (Figure S13). For this system, the energy difference between the triplet and singlet states was strongly coupled with the approaching of O_2 to QND, so it was also possible to represent the reaction pathway as a function of the composite reaction coordinate α. This is done in Figure 6 of the main text, where all the geometries for the intersystem crossing stage appear for α < -0.4 Å. For the ISC stage we only considered the small QM region, and for the sake of simplicity only results with the small QM region are shown in Figure 6.

Calculation of the energy barrier for the intersystem crossing (ISC) step

To compare the effective energy barriers between for two reactions, one spin-allowed (adiabatic) and another spin-forbidden, the barrier height of the latter should be corrected to account for the limited swapping probability between the electronic states involved. According to the Non-Adiabatic Transition State Theory, the rate coefficient of a spin-forbidden transition should be calculated as:[37]

$$k_{Non-Adiabat}(T) = \langle p_{sh} \rangle \, k_{Adiabat}(T), \qquad (1)$$

where $k_{Non\text{-}Adiabat}$ is the actual rate coefficient of the process, $k_{Adiabat}$ would be the rate coefficient of a hypothetical analogous spin-allowed reaction (just one electronic state), and $\langle p_{sh} \rangle$ is the probability of spin-flipping, in this case the probability of hopping from the triplet to the singlet

state at the MECP. $\langle p_{sh} \rangle$ can be calculated from the Landau-Zener theory.[38, 39] Using the double passage version of the spin-diabatic Landau-Zener formula for a given energy is,

$$p_{sh}(E) = (1 - p^{LZ}) + p^{LZ}(1 - p^{LZ}) \quad (2)$$

where p^{LZ} is given by:

$$p^{LZ} = \exp\left(-\frac{2\pi SOC^2}{\hbar \mid \Delta F \mid} \sqrt{\frac{\mu}{2(E - E_{MECP})}} \right) \quad (3)$$

where E is the energy, E_{MECP} is the energy of the MECP, μ is the reduced mass of the normal mode orthogonal to the crossing seam, $|\Delta F|$ is the norm of the difference between the gradients of the two Potential Energy Surfaces at the crossing point, and SOC is the effective spin-orbit coupling calculated at the MECP. For $E < E_{MECP}$, $p^{LZ} = 0$.

SOC was calculated at the CASPT2(22,38)/cc-pvdz level of theory using Open-MOLCAS,[40] with the active space formed by all the valence orbitals of O1, and O2 and those related to the C2-O2 and O1-O2 bonds. The value obtained (SOC= 22 cm^{-1}) was found to be barely sensitive to small changes in the active space. We obtained $\langle p_{sh} \rangle = 0.005$ which leads to an increase of 3.1 kcal/mol to the barrier height.

According to our calculations the energy of the MECP was 10.2 kcal/mol above the energy of the triplet QND^{-} O_2 complex. At the MECP, $r_{C2\text{-}O2}$ =1.62 Å, and the QND moiety is no longer planar. The geometry at the MECP is similar to that obtained by Minaev[41] at the multireference CASSCF(16,11) level of theory. In Ref.[42], Silva computed the MECP for four different (1H)-3-hydroxy-4-oxoquinolines-O_2 complexes, and found considerably different $r_{C2\text{-}O2}$ depending on the substituent, ranging from 2.23 Å for QND to 1.56 Å when the methyl group is replaced by a fluoride. The energy of the MECP for the F substituted oxoquinoline was the smallest, 9.2 kcal/mol, 1 kcal/mol lower than in our calculations, also associated with a small $r_{C2\text{-}O2}$ distance. Differences between our results and those in Ref.[42] can be associated to the different functional used (B3LYP vs M06-2X) or different simulation of the interactions

with the solvent (PCM continuum model using water as a solvent vs QM/MM model). To check the effect of $r_{C2\text{-}O2}$ on the energy of the MECP, we calculated the minimum of the crossing seam for different given $r_{C2\text{-}O2}$ distances and we obtained that for $r_{C2\text{-}O2}$ = 2.0 and 2.3 Å, the energies of the crossing point were only 1.8 kcal/mol and 2.7 kcal/mol above the MECP, respectively.

With respect to the SOC value, a value of 75 cm^{-1} was obtained for other similar spin-forbidden cofactor-less enzymatic reactions,[36] leading to an increase of just 1.6 kcal/mol to the barrier, 1.5 kcal/mol lower than for the reaction between QND and O_2. Assuming that this value is the upper-limit for a cofactor-less reaction involving O_2, other points of the crossing seam (and not necessarily the MECP) could act as effective transition state if their energy is no more than 1.5 kcal/mol above the energy of the MECP and show a SOC value closer to 75 cm^{-1}. Even if that is the case, the effective barrier of the intersystem-crossing stage would be higher than for the CO release stage.

Table S1. Data collection and refinement statistics.

Data collection						
Data set	HOD^{wt}:Xe (30 bar Xe)	HOD^{H251A}:NAA (40 bar O_2 / QND turnover)	HOD^{H251A}:MQO (air)	HOD^{H251A}:MQO (40 bar O_2)	HOD^{S101A}:MQO (air)	HOD^{S101A}:MQO (40 bar O_2)
Beam Line	I24 (DLS)	BM30A (ESRF)	I04-1 (DLS)	I02 (DLS)	I02 (DLS)	ID29 (ESRF)
Wavelength (Å)	0.97981	0.97981	0.91741	0.97949	0.97949	0.97932
Temperature (K)	293	100	100	100	100	100
Resolution range (Å)<br>Highest res. bin (Å)	53.58-2.90<br>(2.95-2.90)	29.12-2.00<br>(2.05-2.00)	44.44-2.00<br>(2.05-2.00)	60.26-2.10<br>(2.16-2.10)	46.56-2.00<br>(2.05-2.00)	60.05-2.00<br>(2.05-2.00)
Space group	$P2_12_12_1$	$P4_1$	$P4_1$	$P4_1$	$P2_12_12_1$	$P4_1$
Cell dimensions (Å)	44.9, 169.5, 169.3	120.1, 120.1, 44.5	119.2, 119.2, 44.4	120.5, 120.5, 44.7	44.8, 167.8, 167.9	120.1, 120.1, 44.8
Unique reflections	29421<br>(1408)	42489<br>(2836)	42360<br>(2876)	37761<br>(9206)	86750<br>(6256)	43233<br>(3144)
Overall redundancy	4.4<br>(4.0)	6.9<br>(2.9)	6.4<br>(4.7)	6.3<br>(3.5)	7.1<br>(7.1)	4.5<br>(4.7)
Completeness, (%)	99.2/91.3<br>(95.8/80.7)	97.9<br>(89.9)	99.3<br>(92.0)	99.4<br>(93.7)	99.8<br>(98.5)	99.0<br>(98.1)
R_{merge}, (%)	19.8<br>(112.7)	7.4<br>(63.9)	10.8<br>(73.7)	9.0<br>(75.8)	9.2<br>(86.6)	15.8<br>(71.1)
R_{pim} (I), (%)	6.1<br>(62.3)	3.3<br>(53.5)	5.0<br>(40.7)	4.1<br>(48.7)	4.0<br>(37.5)	9.3<br>(40.6)
$\langle I/\sigma(I)\rangle$	5.4<br>(1.3)	20.1<br>(1.4)	10.5<br>(1.8)	12.7<br>(1.4)	12.7<br>(2.3)	5.8<br>(1.8)
Wilson B factor ($Å^2$)	59.9	21.2	27.6	28.1	29.6	22.1
**Refinement**						
PDB code	8A97	8OXT	7OJM	7OKZ	8OXN	8ORO
R_{factor} (%)/R_{free}(%)	19.18/22.03	20.30/23.55	20.40/23.67	19.02/22.84	17.34/19.42	22.74/26.87
Twin law	*-h, -l, -k*	*-k, -h, -l*	*-k, -h, -l*	*-k, -h, -l*	*-h, -l, -k*	*-k, -h, -l*
Twin fraction	0.52/0.48	0.85/0.15	0.84/0.16	0.88/0.12	0.53/0.47	0.84/0.16
# non-H atoms	9096	4791	4851	4867	9196	5018
rms bond lengths (Å)	0.008	0.007	0.005	0.006	0.006	0.005
rms bond angles (°)	1.31	1.39	1.33	1.32	1.34	1.28

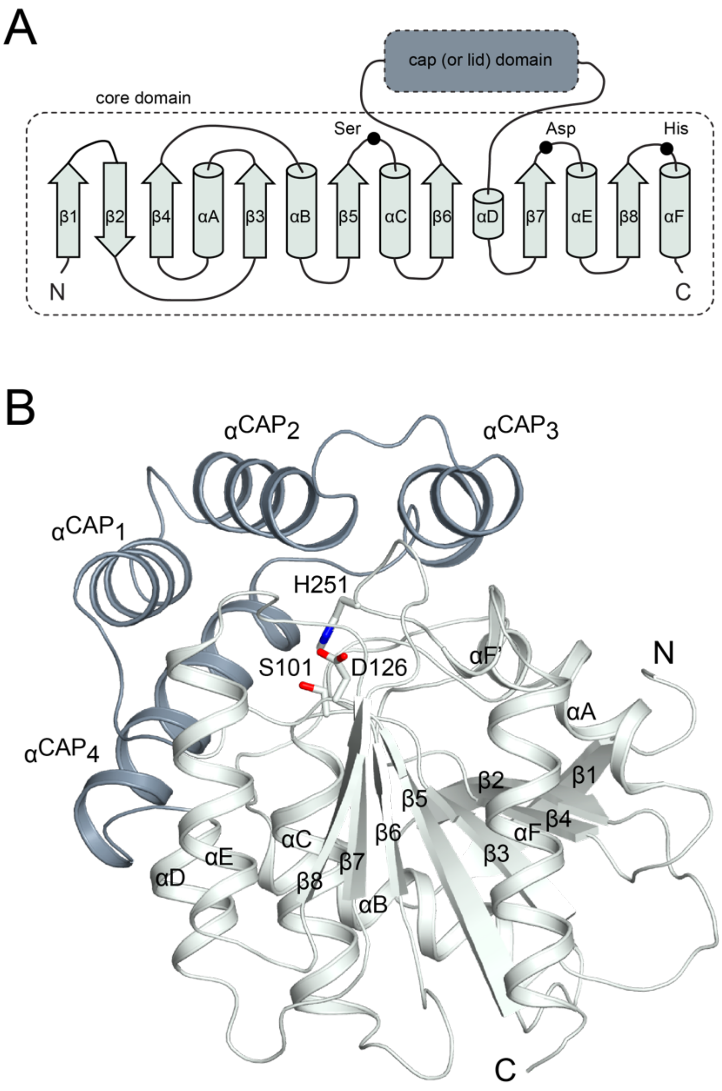

Figure S1. ABH fold. (A) General ABH fold topology and (B) ABH three-dimensional architecture exemplified by the crystal structure of *Arthrobacter nitroguajacolicus* Rü61a 1-*H*-3-hydroxy-4-oxoquinaldine 2,4-dioxygenase (HOD) (PDB code 2WJ3). HOD is the focus of the present study. The core domain of ABHs (here shown in light grey) is an eight-stranded, mostly parallel, β-sheet surrounded by α-helices. This is very often embellished by a cap or lid domain (here shown in dark grey) inserted between β6 and the short αD that provides structural and functional variation. In the case of HOD the cap domain is constituted by four α-helices. A very conserved feature of ABHs is the Ser (nucleophile), His, and Asp (acidic residue, sometimes replaced by Asn) catalytic triad represented by black dots in the topology diagram. S101, H251 and D126 form the catalytic triad in HOD. These residues are shown as sticks in panel B with nitrogen and oxygen atoms in blue and red, respectively. The nucleophile is hosted by the 'nucleophilic elbow', a tight turn connecting β5 and αC. In some ABH-fold dioxygenases the nucleophile is replaced by an alanine. The acidic residue, generally positioned after β7, is relocated after β6 in HOD. The catalytic histidine is near the C-terminus after the β8 strand. N and C indicate the protein N-terminus and C-terminus, respectively.

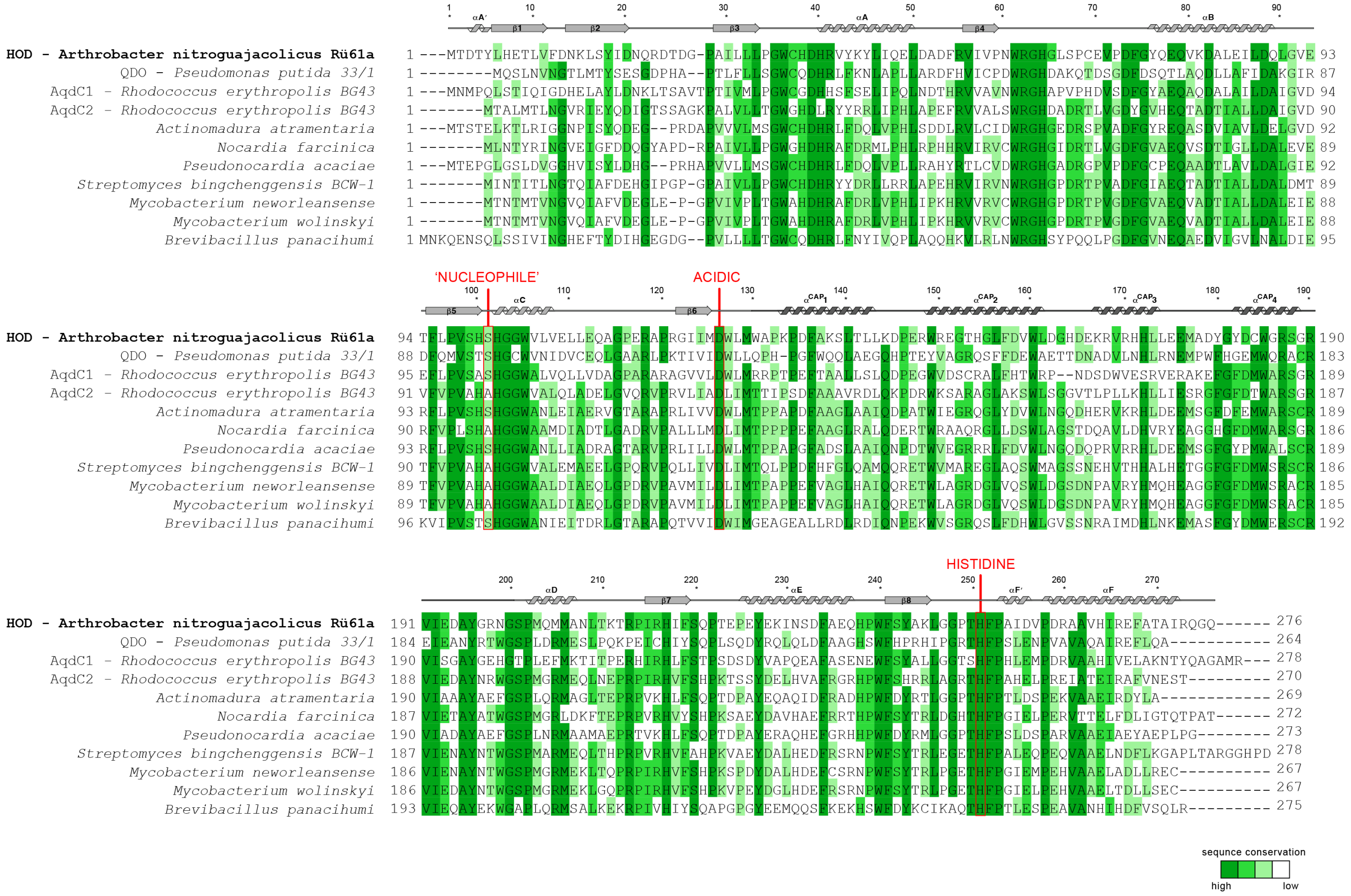

Figure S2. Sequence alignment of various predicted and validated bacterial ABH-fold cofactor-independent 2,4-dioxygenases exhibiting high similarity to *A. nitroguajacolicus* Rü61a 1-*H*-3-hydroxy-4-oxoquinaldine 2,4-dioxygenase (HOD). Residues are highlighted in different shades of green according to their sequence identity. Secondary structure elements for HOD are also shown for reference. Residues of the nucleophile-histidine-acidic catalytic triad are indicated. Whilst the His-Asp dyad is totally conserved, the nucleophile is not and, in several organisms, the latter residues is replaced by an alanine.

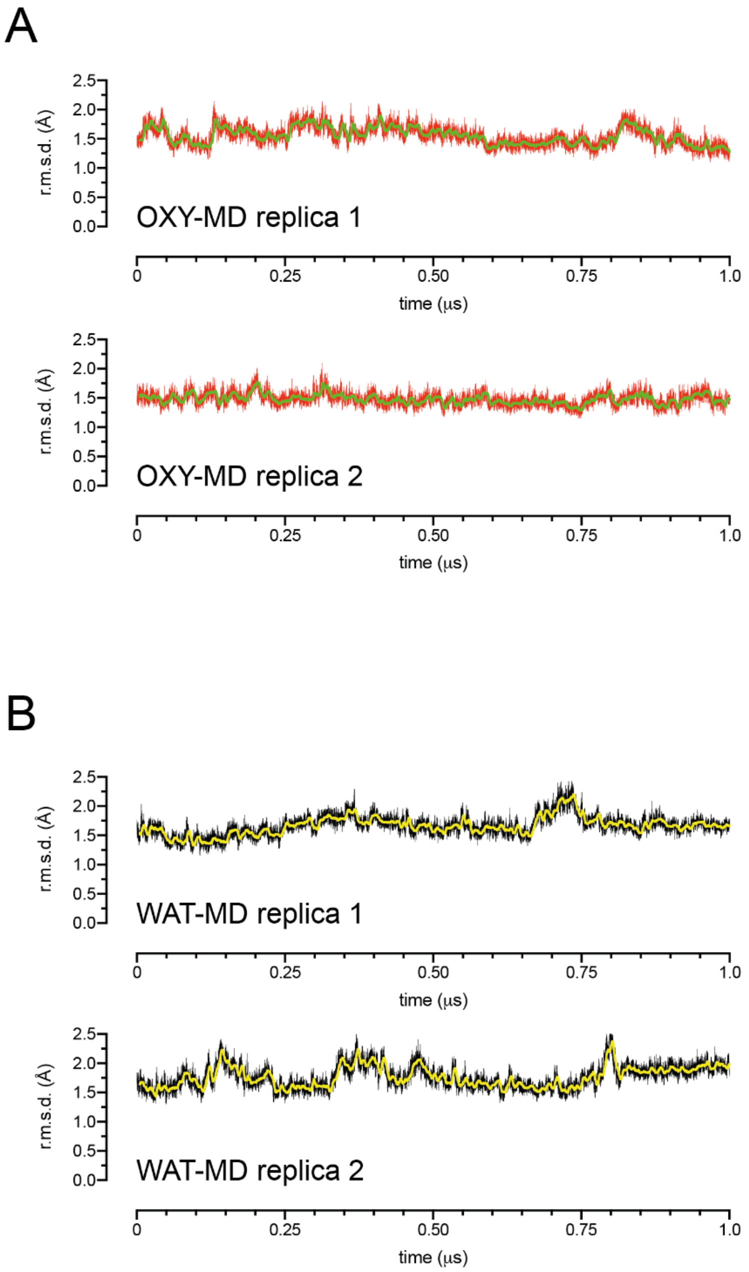

Figure S3. Root mean square deviation (r.m.s.d.) from the starting equilibrated crystallographic structure for HOD during 1 μs-long replicas Molecular Dynamics (MD) simulations. OXY-MD runs (A) were carried out in the presence of ten O_2 molecules in a solvated box of approximately 74×74×74 Å^3. WAT-MD did not include O_2 molecules in the simulations. Sampling was performed every 50 ps of each run. Thick lines represent the moving average calculated using a 5 ns window.

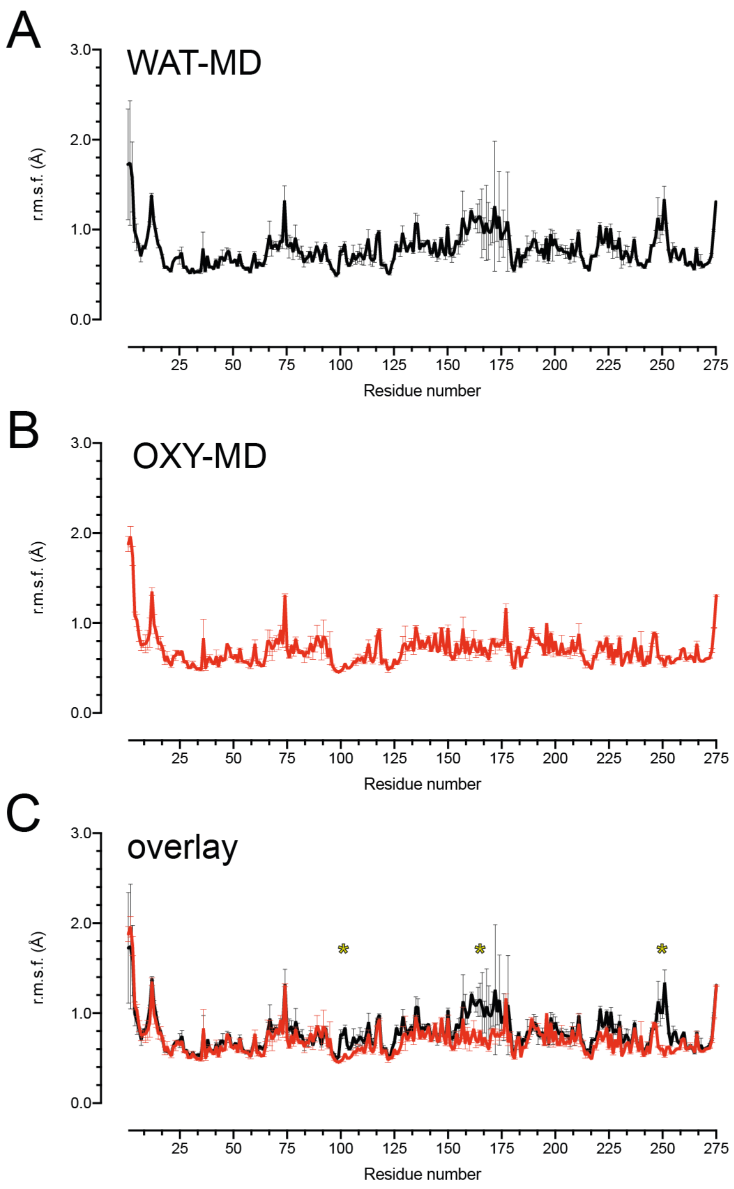

Figure S4. Root mean square fluctuations (r.m.s.f.) from the average structure for HOD during WAT-MD (A) and OXY-MD (B) simulations. OXY-MD runs were carried out in the presence of ten O_2 molecules to a solvated box of approximately 74×74×74 Å^3. WAT-MD did not include O_2 molecules in the simulations. Two replica 1-µs simulations were run for each system and data are presented as average r.m.s.f. with standard deviations. (C) is an overlay of A and B with asterisks highlighting the regions with different mobility.

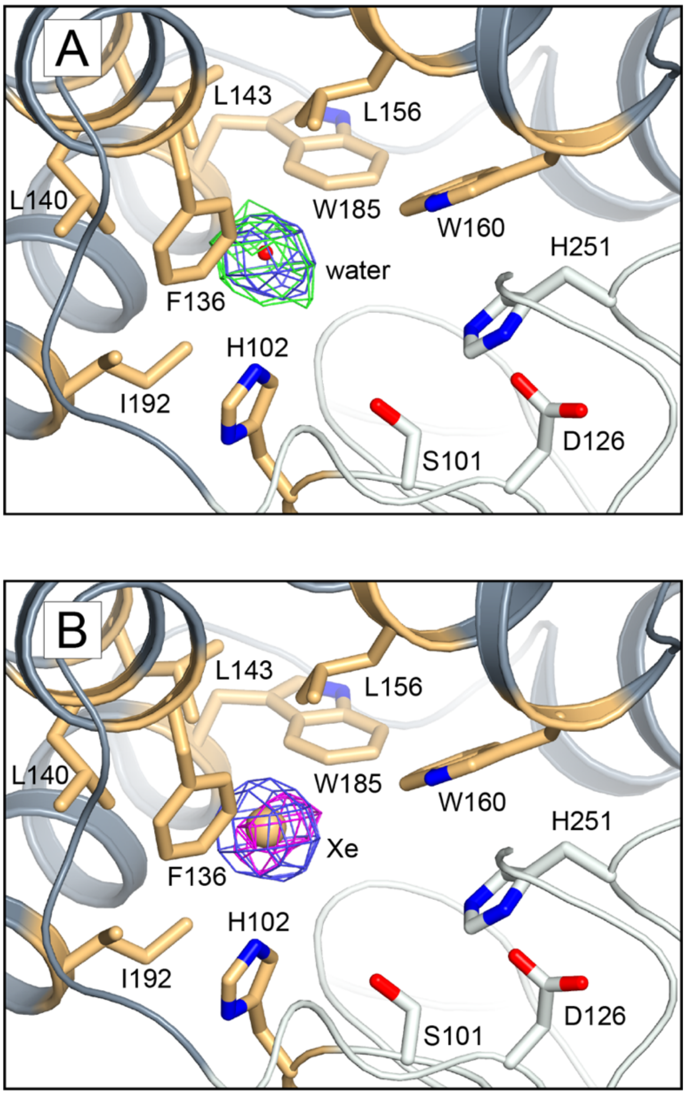

Figure S5. Validation of Xe binding at the *B*-site. (A) Interpretation of the Xe site as a water molecule results in significant residual density in Fourier difference maps. Post-refinement $2mF_o$-DF_c and mF_o-DF_c electron density maps are shown at the +1.0σ (blue) and +3.0σ (green) levels, respectively. (B) Interpretation as a Xe is satisfactory and occupancies refine between 0.4 and 0.6 in the four different molecules in the a.u. The $2mF_o$-DF_c electron density map is shown at the +1.0σ level in blue. The map displayed in magenta is the NCS-averaged anomalous difference map contoured at +5.5σ. We note that the anomalous signal for this experiment is weak as the wavelength used for data collection was far from the f" peak to minimize radiation damage at room temperature. Also, data at 2.9 Å-resolution exhibit non-merohedral twinning (twinning fractions 0.52/0.48) which negatively affects the anomalous signal. Despite these limitations the interpretation of this site as Xe is unambiguous. Hydrophobic residues stabilizing the Xe atom are highlighted in gold and shown as sticks. The catalytic triad (S101/H251/D126) is also shown.

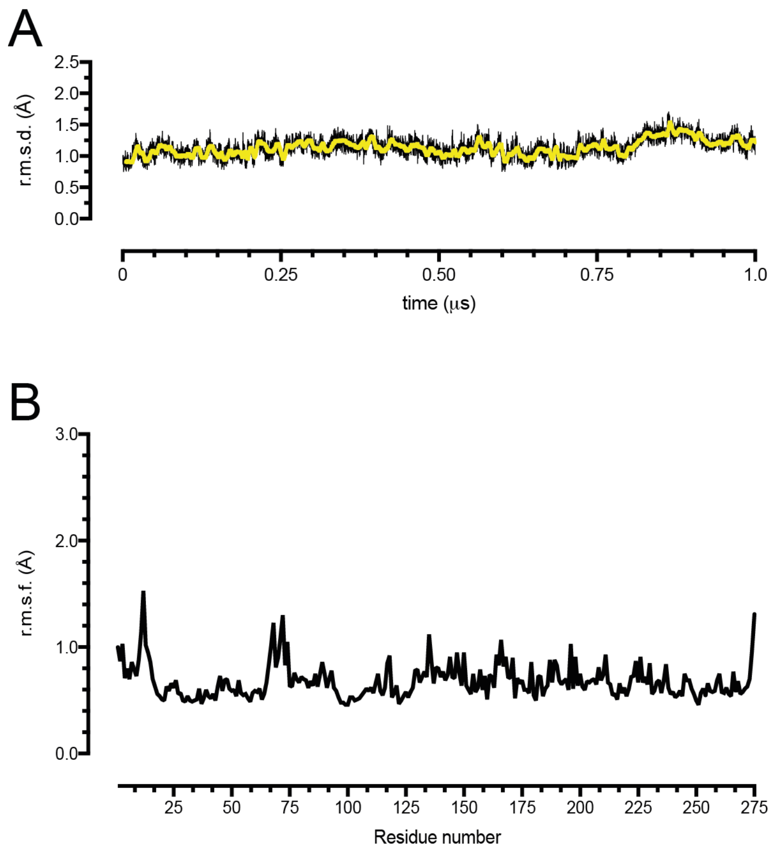

Figure S6. Molecular Dynamics (MD) simulation of the HOD-QND complex. (A) Root mean square deviation (r.m.s.d.) from the starting equilibrated crystallographic structure for the HOD-QND complex (PDB code 2WJ4), during a 1 μs-long MD simulation. Sampling was performed every 50 ps of each run. The thick yellow line lines represent the moving average calculated using a 5 ns window; (B) root mean square fluctuation (r.m.s.f.) from the average structure during the simulation.

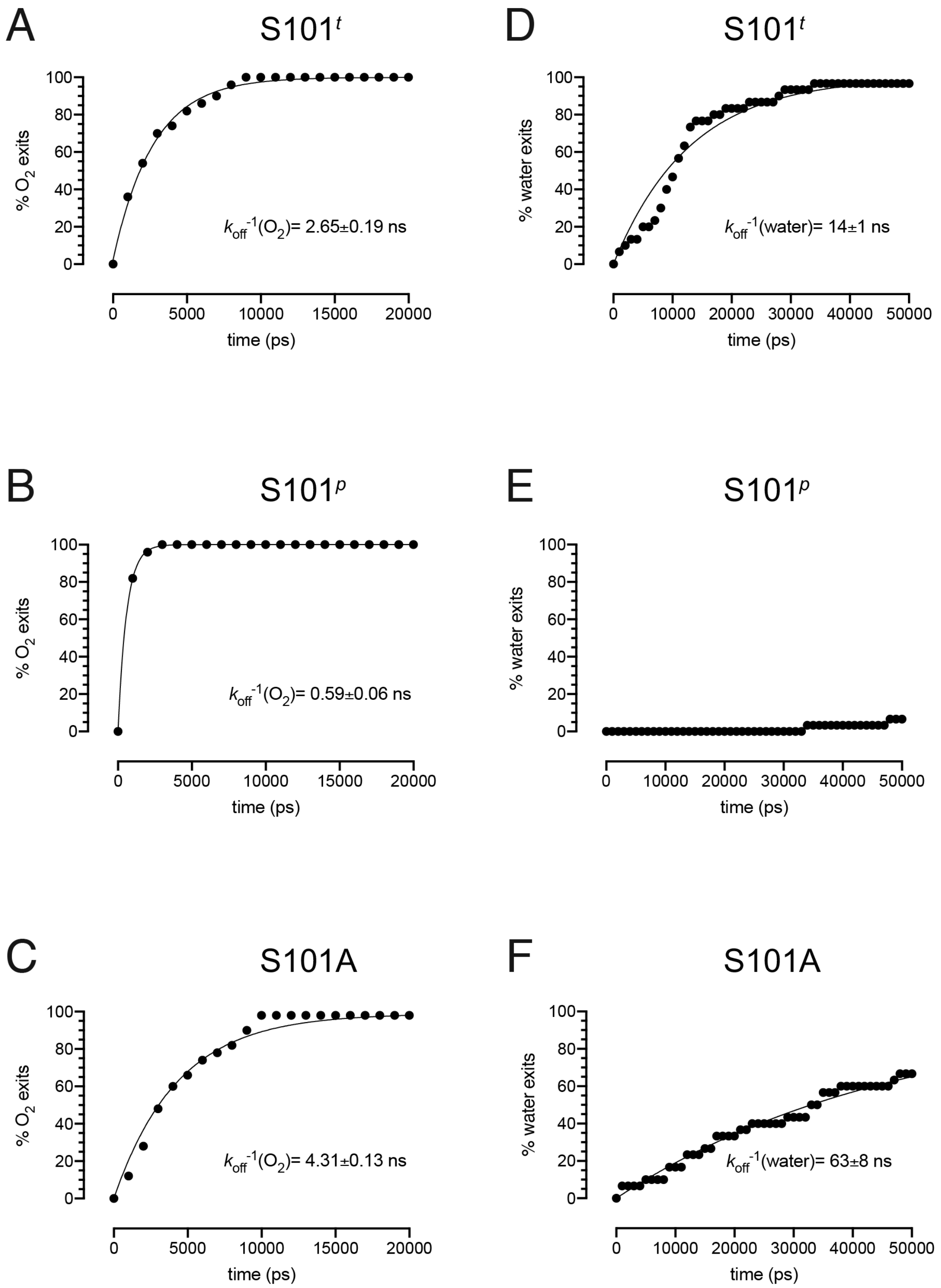

Figure S7. Fitting of residence times for O_2 (A-C) or water (D-F) at the *R*-site with S101 restrained to either the *trans* rotamer (A,D), or to the *plus* rotamer (B,E), or, alternatively, with S101 is replaced by an alanine (C,F). The cumulative distributions were fitted to the equation (2) to determine the residence times ($\lambda = k_{\text{off}}^{-1}$). In the case of (E) fitting is not possible as there are too few escape events with 50 ns.

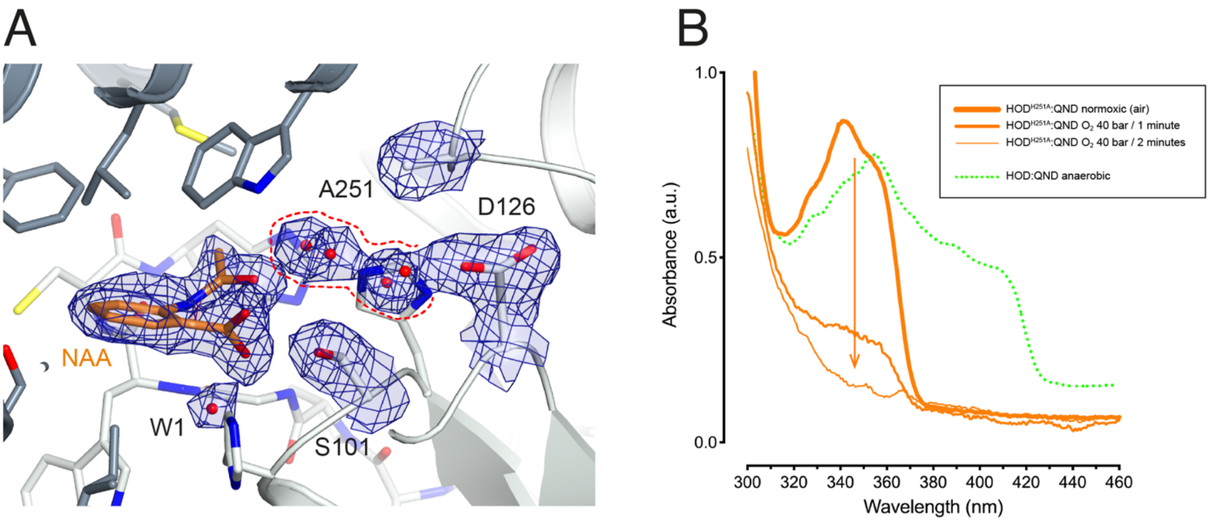

Figure S8. *In crystallo* turnover. (A) Active site of the HOD^{H251A}-QND complex following O_2 pressurization at 40 bar for 2 minutes. QND is fully converted to *N*-acetylanthranilate (NAA), the product of the HOD-catalyzed reaction. Electron density maps ($2mF_o$-DF_c) are shown at the +1.0σ level for NAA, the side chains of S101, D126, A251 (catalytic triad), the water molecule at the *R*-site (W1) and the solvent network (highlighted by the red dotted line) that connects D126 to the organic ligand. (B) *in crystallo* UV-visible spectra of HOD^{H251A}-QND under normoxic conditions (thick orange line), after 1-minute O_2 pressurization (medium thickness orange line), after 2-minutes O_2 pressurization (thon orange line). The *in crystallo* UV-visible spectrum of the anaerobic HOD-QND complex (dotted green line) is also shown for reference. A small offset was added to the green trace to improve clarity. Pressurization enables O_2 access to the *R*-site in the pre-formed HOD^{H251A}-QND complex promoting turnover without buildup of the substrate anion. The structure suggests that the marginal residual catalytic activity of the HOD^{H251A} variant (k_{cat} = 0.0006 s^{-1} at pH 6.5 compared to 16.3 s^{-1} for wtHOD) relies on solvent-mediated substrate deprotonation. UV/Vis spectra were recorded using the online microspec at the BM30A beamline at the ESRF.

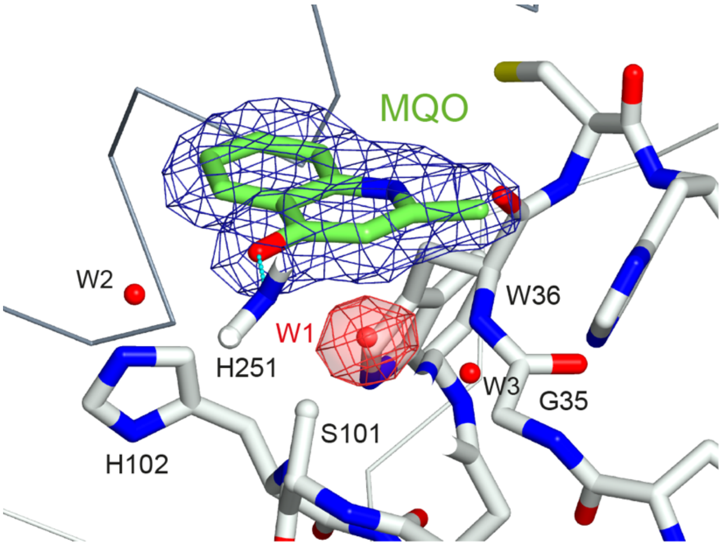

Figure S9. Representative $2mF_o$-DF_c electron density for the HOD^{S101A}-MQO complex under normoxic conditions. Electron density maps are shown at the +1.0σ level as chicken-wire representation for MQO (blue) and a water molecule (W1) at the *R*-site. The latter is displayed in red for clarity. The view is the same as in Figure 5B of the main text.

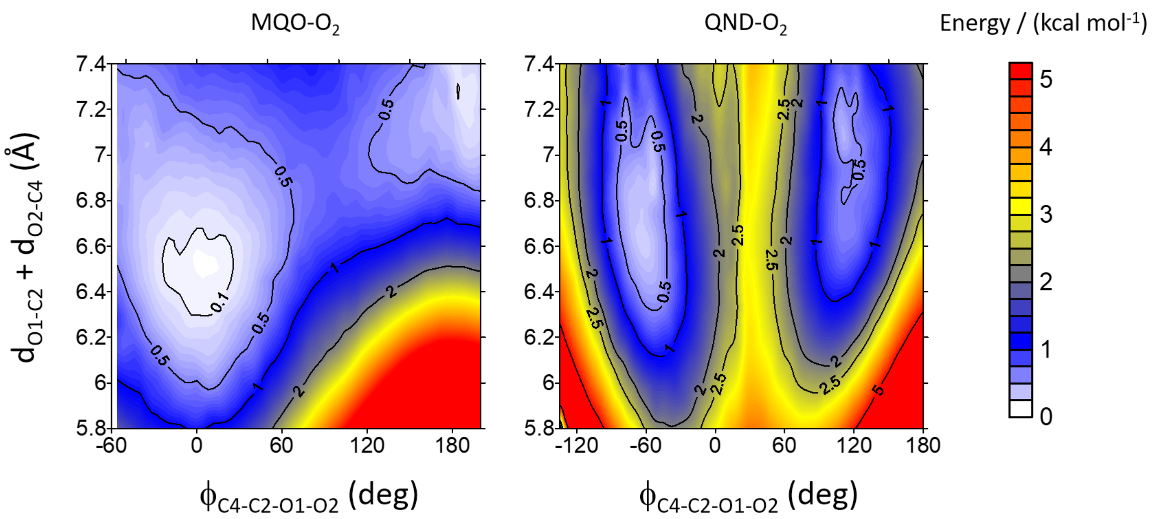

Figure S10. 3D contour maps for the energy profiles for the O_2-ligand (MQO on the left and QND on the right) complexes as a function of the dihedral angle Φ = (C4-C2-O2-O1), and the sum of (C2-O1) and (C4-O2) distances. The latter was used as a restraint in the calculations for the isolated complexes. Around the equilibrium geometries, the energy is relatively insensitive to small changes in the restraint value. The two minima for the MQO-O_2 complex at Φ=0 and 180º represents the same structure, reflecting that O1 and O2 are equivalent.

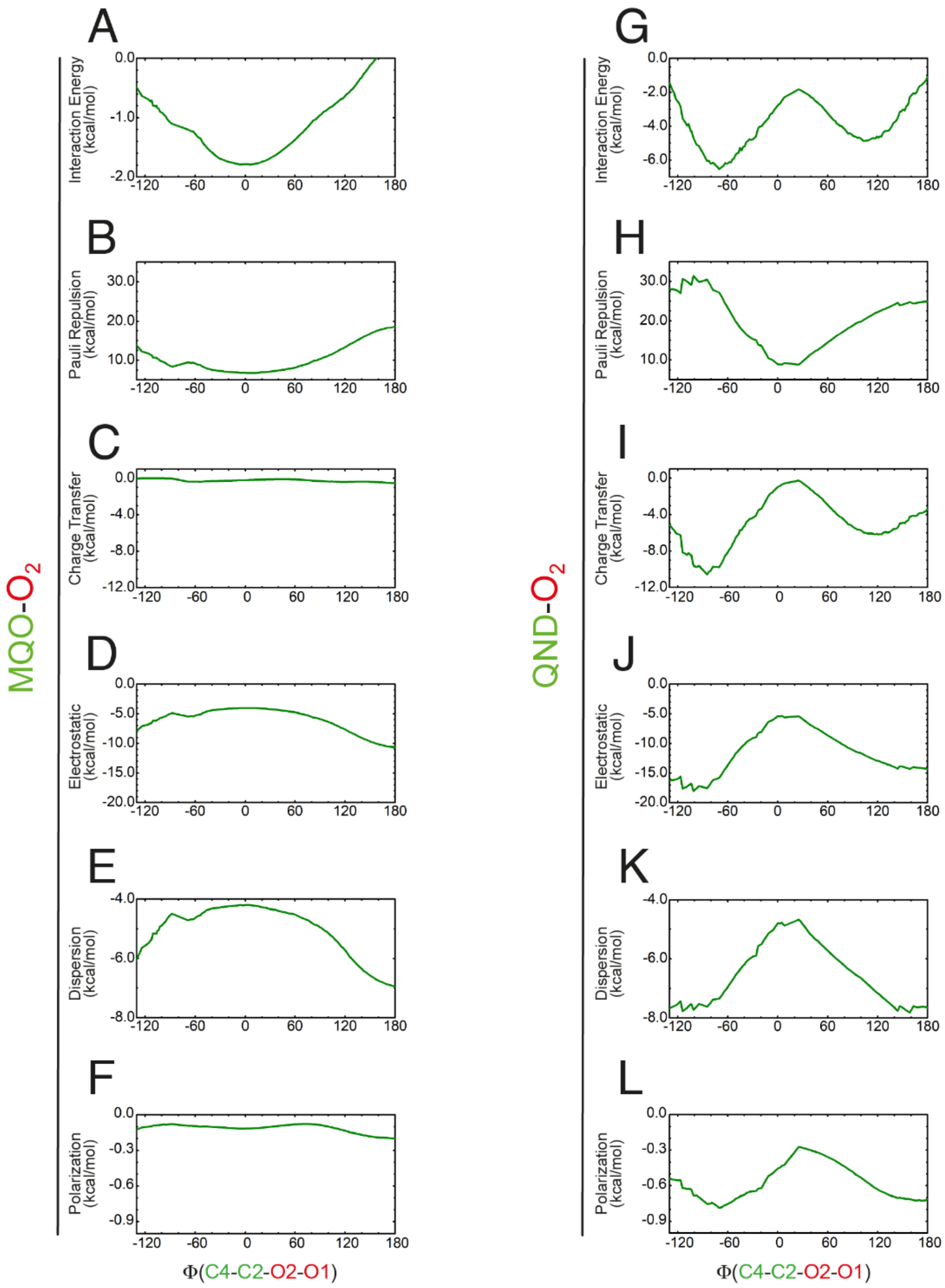

Figure S11. Energy decomposition analysis for the MQO-O_2 (A-F) and QND-O_2 (G-L) complexes as a function of the dihedral angle Φ=(C4-C2-O2-O1). Interaction energy values (A,B) are the sum of their respective Pauli repulsion (B,H), charge transfer (C,I), electrostatic (D,J), dispersion (E,K), and polarization (F,L) terms (A=B+C+D+E+F, G=H+I+J+K+L).

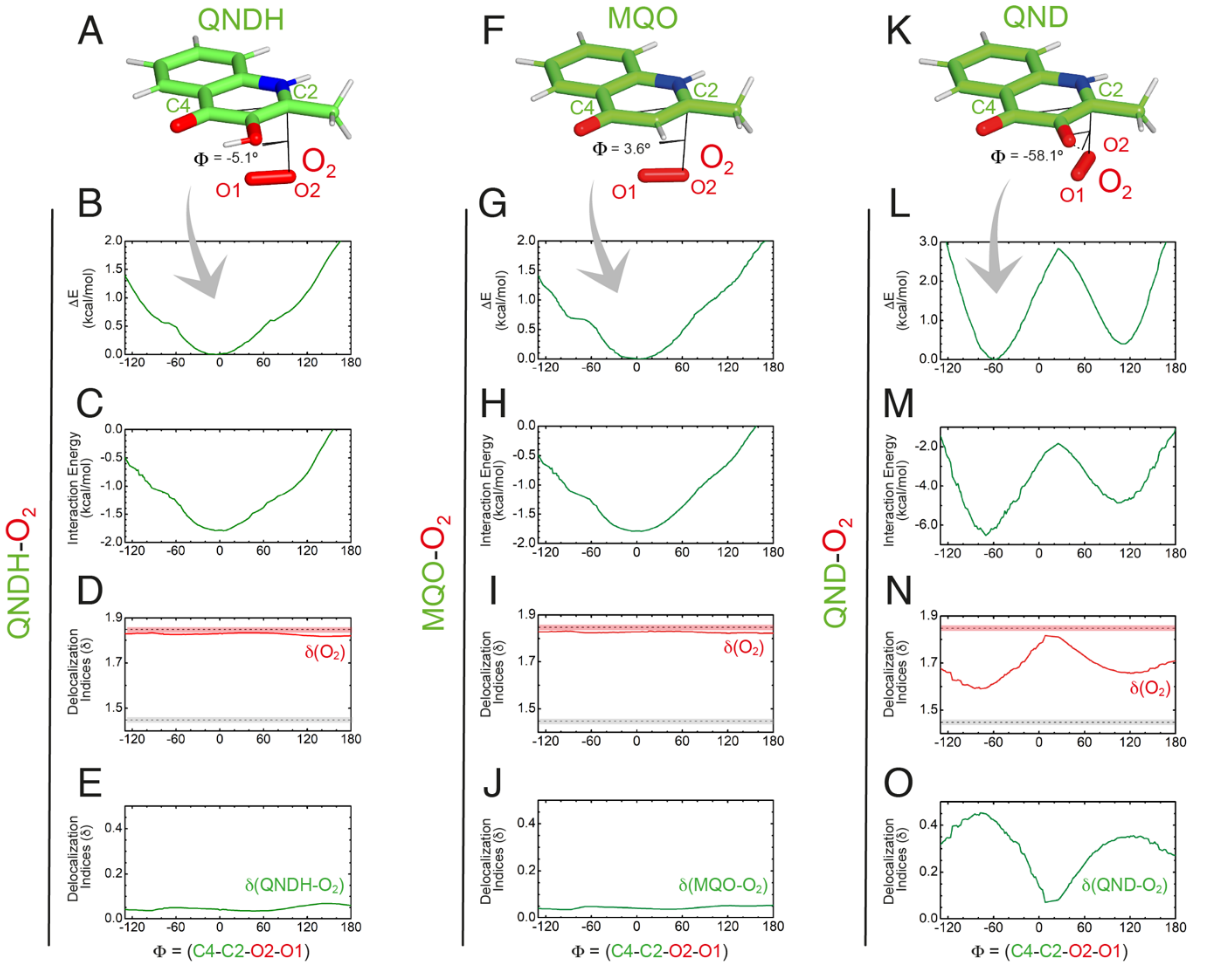

Figure S12. QM geometry restrained optimization and delocalization indices for QNDH-O_2 (A-E), MQO-O_2 (F-J) and QND-O_2 (K-O) complexes as a function of the dihedral angle Φ = (C4-C2-O2-O1). Panels (F-O) are identical to panels (C-L) in Figure 5 of the main text and are shown here for reference. In panels (D,I,N) delocalization indices for isolated O_2 (dashed red line) and the aromatic C-C bond in benzene (dashed grey line) are given for reference. Protonation of QND (QNDH) results in an overall neutral system QNDH-O_2 system that behaves like MQO-O_2.

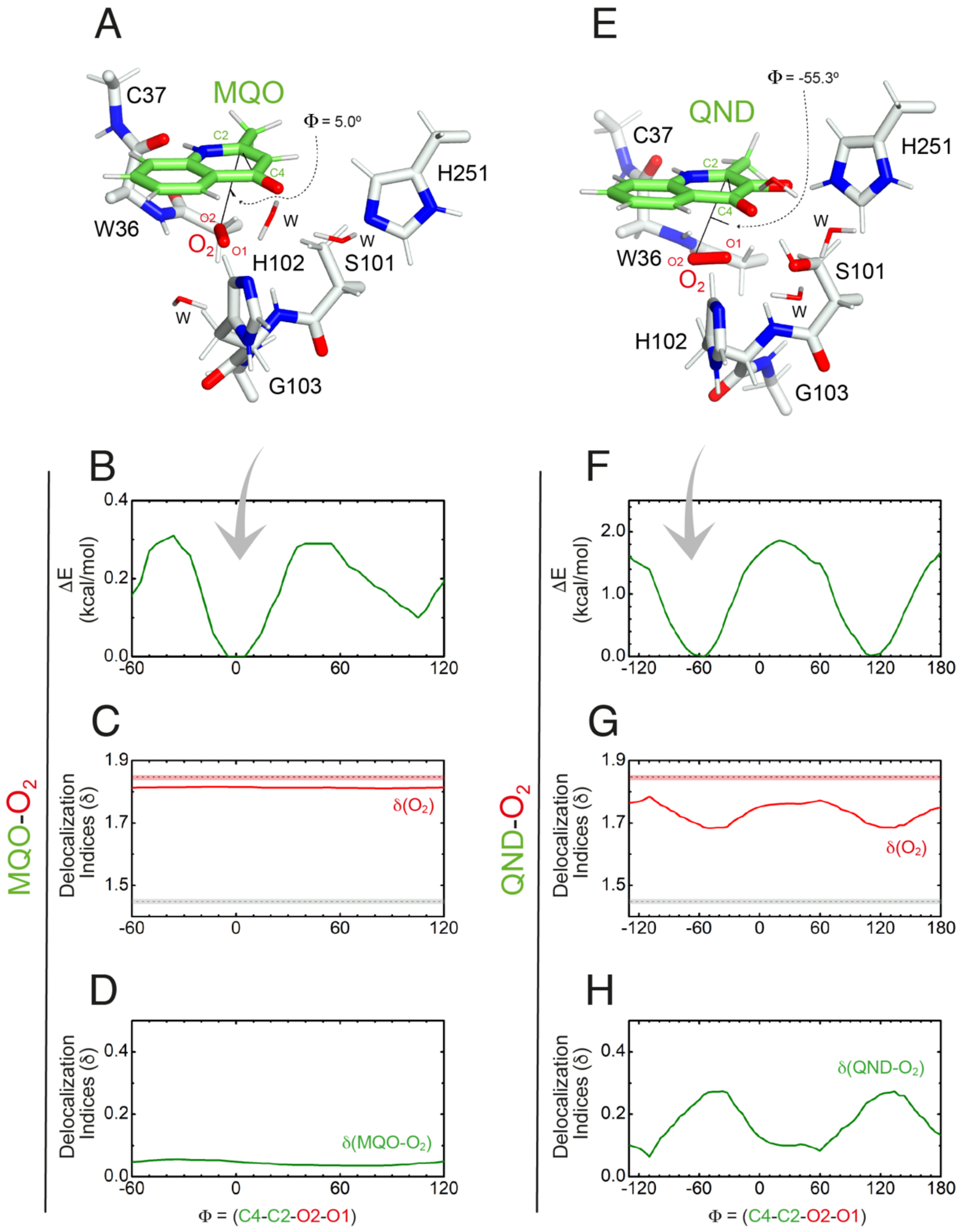

Figure S13. QM/MM geometry restrained optimization and delocalization indices for the MQO-O_2 (A-D) and QND-O_2 (E-H) complexes as a function of the dihedral angle Φ = (C4-C2-O2-O1). (A,E) are stick representations of the minimized structures. (B,F) show the total energy of the complexes as a function of Φ. (C,G) display the delocalization index (δ) for the O-O bond in the complex. For comparison, the dashed red and grey lines represent the delocalization indices for O-O and C-C bonds in isolated O_2 and in benzene, respectively. (D,H) show the delocalization index for the interaction between the substrate and O_2.

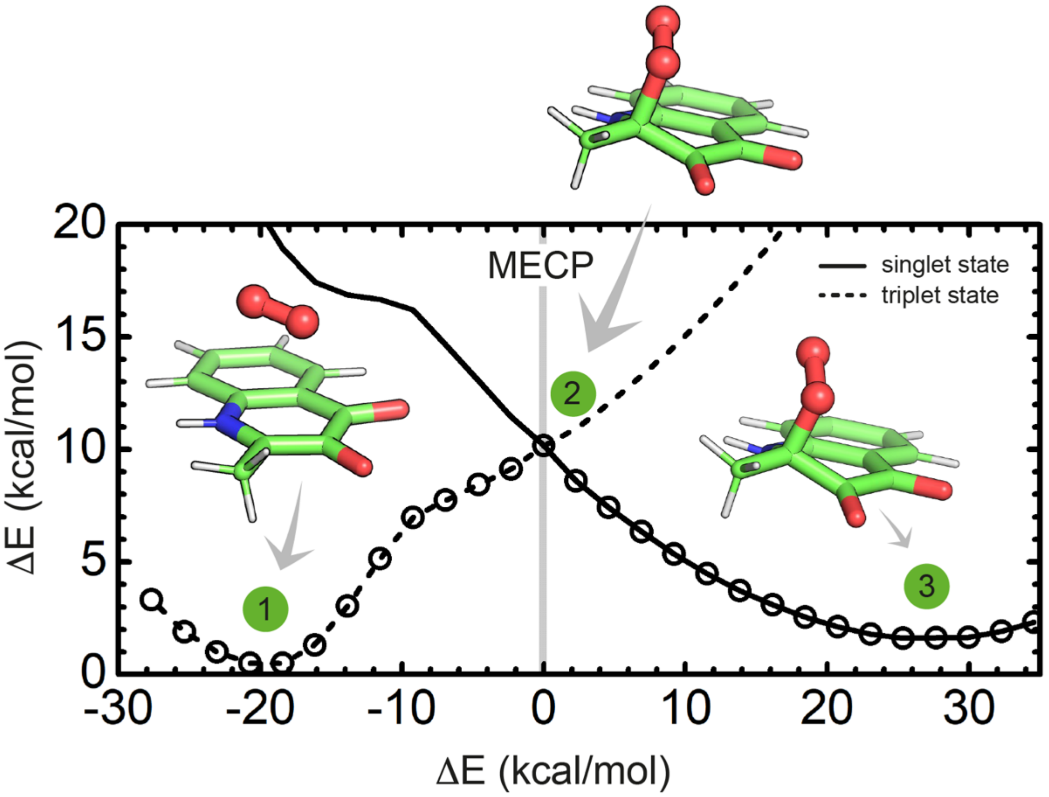

Figure S14. Energy profile for the transition from the triplet to the singlet state and formation of the peroxide intermediate. The energy is presented as a function of the energy difference between the triplet and the singlet state, which was chosen as a reaction coordinate. The relevant structures at the points indicated are shown as stick (ball-and-stick for O_2) representations. Structure 2 represents the minimum energy crossing point (MECP).

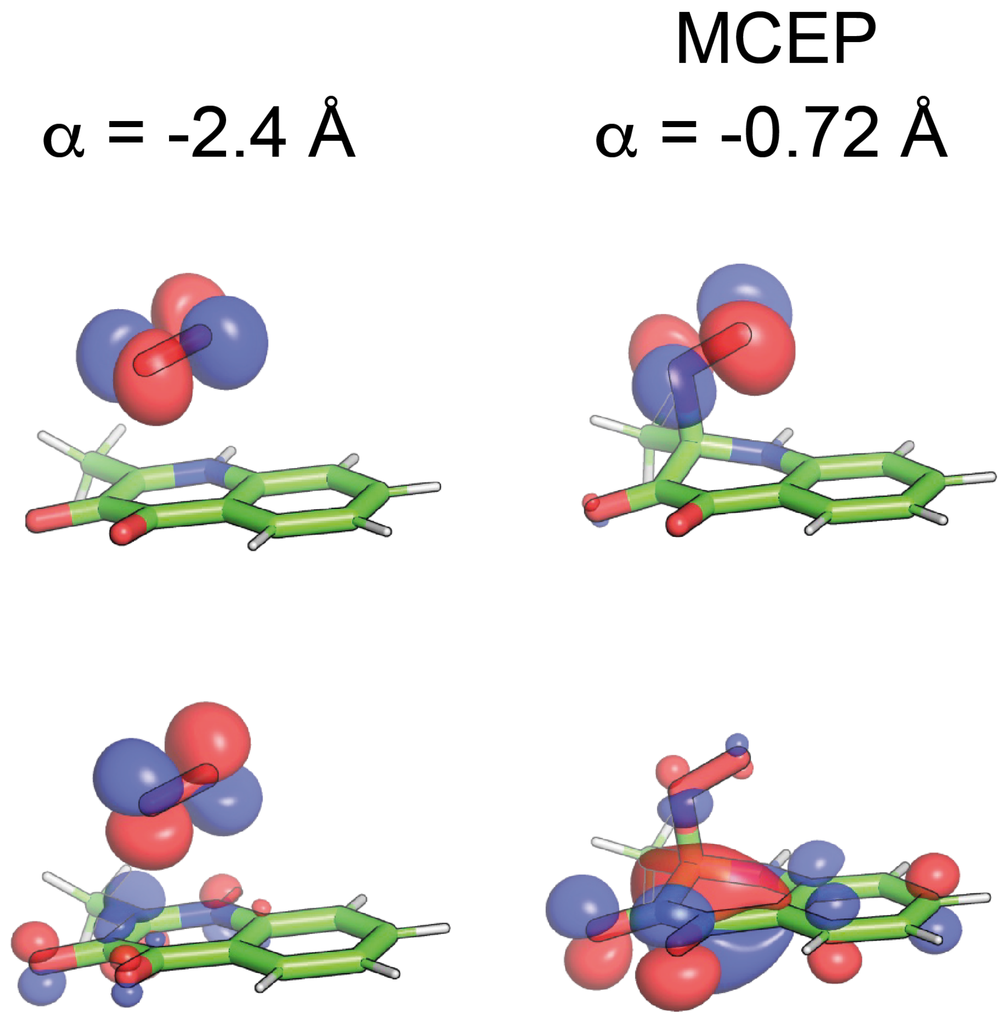

Figure S15. Shape of the half-occupied orbitals for the QND-O_2 complex at the reactants asymptote (left panels), and at the MECP (right panels). The orbitals were localized following the procedure described in Ref. [43] At the reactants asymptote, half-occupied orbitals correspond to the two π* orbitals of O_2. The π* perpendicular to the π cloud of QND (π_x*), is not mixed with any QND orbital. However, the π* orbital that stacks with the π cloud of QND (π_y*) is significantly mixed with the π cloud of QND. As QND and O_2 approach each other, mixing is more substantial, leading to an occupied orbital with bonding character along the C2-O bond, and a higher energy orbital that shows an antibonding character both along O1-O2 and C2-O bonds. The latter orbital is singly occupied in the triplet state but it is a virtual orbital in the singlet state, explaining why the singlet state is stabilized along the reaction path while triplet state is destabilized.

References

1. R. A. Steiner, U. Frerichs-Deeken and S. Fetzner, Crystallization and preliminary X-ray analysis of 1H-3-hydroxy-4-oxoquinaldine 2,4-dioxygenase from Arthrobacter nitroguajacolicus Ru61a: a cofactor-devoid dioxygenase of the alpha/beta-hydrolase-fold superfamily, *Acta Crystallogr Sect F Struct Biol Cryst Commun*, 2007, **63**, 382-385.
2. B. Lafumat, C. Mueller-Dieckmann, G. Leonard, N. Colloc'h, T. Prangé, T. Giraud, F. Dobias, A. Royant, P. van der Linden and P. Carpentier, Gas-sensitive biological crystals processed in pressurized oxygen and krypton atmospheres: deciphering gas channels in proteins using a novel `soak-and-freeze' methodology, *J Appl Cryst*, 2016, **49**, 1478-1487.
3. G. Winter, xia2: an expert system for macromolecular crystallography data reduction, *J Appl Cryst*, 2010, **43**, 186-190.
4. A. J. McCoy, R. W. Grosse-Kunstleve, P. D. Adams, M. D. Winn, L. C. Storoni and R. J. Read, Phaser crystallographic software, *J Appl Crystallogr*, 2007, **40**, 658-674.
5. G. N. Murshudov, P. Skubak, A. A. Lebedev, N. S. Pannu, R. A. Steiner, R. A. Nicholls, M. D. Winn, F. Long and A. A. Vagin, REFMAC5 for the refinement of macromolecular crystal structures, *Acta Crystallogr D Biol Crystallogr*, 2011, **67**, 355-367.
6. P. Emsley and K. Cowtan, Coot: model-building tools for molecular graphics, *Acta Crystallogr D Biol Crystallogr*, 2004, **60**, 2126-2132.
7. A. Hernandez-Ortega, M. G. Quesne, S. Bui, D. P. Heuts, R. A. Steiner, D. J. Heyes, S. P. de Visser and N. S. Scrutton, Origin of the proton-transfer step in the cofactor-free (1H)-3-hydroxy-4-oxoquinaldine 2,4-dioxygenase: effect of the basicity of an active site His residue, *J Biol Chem*, 2014, **289**, 8620-8632.
8. R. A. Steiner, H. J. Janssen, P. Roversi, A. J. Oakley and S. Fetzner, Structural basis for cofactor-independent dioxygenation of N-heteroaromatic compounds at the alpha/beta-hydrolase fold, *Proc Natl Acad Sci U S A*, 2010, **107**, 657-662.
9. D. von Stetten, T. Giraud, P. Carpentier, F. Sever, M. Terrien, F. Dobias, D. H. Juers, D. Flot, C. Mueller-Dieckmann, G. A. Leonard, D. de Sanctis and A. Royant, In crystallo optical spectroscopy (icOS) as a complementary tool on the macromolecular crystallography beamlines of the ESRF, *Acta Crystallogr D Biol Crystallogr*, 2015, **71**, 15-26.
10. D. A. Case, T. E. Cheatham, 3rd, T. Darden, H. Gohlke, R. Luo, K. M. Merz, Jr., A. Onufriev, C. Simmerling, B. Wang and R. J. Woods, The Amber biomolecular simulation programs, *J Comput Chem*, 2005, **26**, 1668-1688.
11. M. H. Olsson, C. R. Sondergaard, M. Rostkowski and J. H. Jensen, PROPKA3: Consistent Treatment of Internal and Surface Residues in Empirical pKa Predictions, *J Chem Theory Comput*, 2011, **7**, 525-537.
12. C. R. Sondergaard, M. H. Olsson, M. Rostkowski and J. H. Jensen, Improved Treatment of Ligands and Coupling Effects in Empirical Calculation and Rationalization of pKa Values, *J Chem Theory Comput*, 2011, **7**, 2284-2295.
13. J. C. Phillips, R. Braun, W. Wang, J. Gumbart, E. Tajkhorshid, E. Villa, C. Chipot, R. D. Skeel, L. Kalé and K. Schulten, Scalable molecular dynamics with NAMD, *J. Comput. Chem*, 2005, **26**, 1781-1802.
14. M. J. Harvey, G. Giupponi and G. D. Fabritiis, ACEMD: Accelerating Biomolecular Dynamics in the Microsecond Time Scale, *J Chem Theory Comput*, 2009, **5**, 1632-1639.

15. U. Essmann, L. Perera and M. L. Berkowitz, A smooth particle mesh Ewald method, *J. Chem. Phys*, 1995, **103**, 8577.
16. K. Lindorff-Larsen, S. Piana, K. Palmo, P. Maragakis, J. L. Klepeis, R. O. Dror and D. E. Shaw, Improved side-chain torsion potentials for the Amber ff99SB protein force field, *Proteins*, 2010, **78**, 1950-1958.
17. W. L. Jorgensen, J. Chandrasekhar and J. D. Madura, Comparison of simple potential functions for simulating liquid water, *J. Chem. Phys*, 1983, **79**, 926.
18. J. Wang, R. M. Wolf, J. W. Caldwell, P. A. Kollman and D. A. Case, Development and testing of a general amber force field, *J Comput Chem*, 2004, **25**, 1157-1174.
19. C. I. Bayly, P. Cieplak, W. Cornell and P. A. Kollman, A well-behaved electrostatic potential based method using charge restraints for deriving atomic charges: the RESP model, *J. Phys. Chem.*, 1993, **97**, 10269–10280.
20. M. J. Frisch, G. W. Trucks, H. B. Schlegel, G. E. Scuseria, M. A. Robb, J. R. Cheeseman, G. Scalmani, V. Barone, G. A. Petersson, H. Nakatsuji, X. Li, M. Caricato, A. V. Marenich, J. Bloino, B. G. Janesko, R. Gomperts, B. Mennucci, H. P. Hratchian, J. V. Ortiz, A. F. Izmaylov, J. L. Sonnenberg, Williams, F. Ding, F. Lipparini, F. Egidi, J. Goings, B. Peng, A. Petrone, T. Henderson, D. Ranasinghe, V. G. Zakrzewski, J. Gao, N. Rega, G. Zheng, W. Liang, M. Hada, M. Ehara, K. Toyota, R. Fukuda, J. Hasegawa, M. Ishida, T. Nakajima, Y. Honda, O. Kitao, H. Nakai, T. Vreven, K. Throssell, J. A. Montgomery Jr., J. E. Peralta, F. Ogliaro, M. J. Bearpark, J. J. Heyd, E. N. Brothers, K. N. Kudin, V. N. Staroverov, T. A. Keith, R. Kobayashi, J. Normand, K. Raghavachari, A. P. Rendell, J. C. Burant, S. S. Iyengar, J. Tomasi, M. Cossi, J. M. Millam, M. Klene, C. Adamo, R. Cammi, J. W. Ochterski, R. L. Martin, K. Morokuma, O. Farkas, J. B. Foresman and D. J. Fox, Gaussian 16 Rev. A.03*Journal*, 2016.
21. M. Ceccarelli, P. Procacci and M. Marchi, An ab initio force field for the cofactors of bacterial photosynthesis, *J Comput Chem*, 2003, **24**, 129-142.
22. M. Ceccarelli, R. Anedda, M. Casu and P. Ruggerone, CO escape from myoglobin with metadynamics simulations, *Proteins*, 2008, **71**, 1231-1236.
23. A. Barducci, G. Bussi and M. Parrinello, Well-tempered metadynamics: a smoothly converging and tunable free-energy method, *Phys Rev Lett*, 2008, **100**, 020603.
24. P. Tiwary and M. Parrinello, From metadynamics to dynamics, *Phys Rev Lett*, 2013, **111**, 230602.
25. R. Cabriolu, K. M. Skjelbred Refsnes, P. G. Bolhuis and T. S. van Erp, Foundations and latest advances in replica exchange transition interface sampling, *J Chem Phys*, 2017, **147**, 152722.
26. Y. Shao, Z. Gan, E. Epifanovsky, A. T. B. Gilbert, M. Wormit, J. Kussmann, A. W. Lange, A. Behn, J. Deng, X. Feng, D. Ghosh, M. Goldey, P. R. Horn, L. D. Jacobson, I. Kaliman, R. Z. Khaliullin, T. Kuś, A. Landau, J. Liu, E. I. Proynov, Y. M. Rhee, R. M. Richard, M. A. Rohrdanz, R. P. Steele, E. J. Sundstrom, H. L. Woodcock, P. M. Zimmerman, D. Zuev, B. Albrecht, E. Alguire, B. Austin, G. J. O. Beran, Y. A. Bernard, E. Berquist, K. Brandhorst, K. B. Bravaya, S. T. Brown, D. Casanova, C.-M. Chang, Y. Chen, S. H. Chien, K. D. Closser, D. L. Crittenden, M. Diedenhofen, R. A. DiStasio, H. Do, A. D. Dutoi, R. G. Edgar, S. Fatehi, L. Fusti-Molnar, A. Ghysels, A. Golubeva-Zadorozhnaya, J. Gomes, M. W. D. Hanson-Heine, P. H. P. Harbach, A. W. Hauser, E. G. Hohenstein, Z. C. Holden, T.-C. Jagau, H. Ji, B. Kaduk, K. Khistyaev, J. Kim, J. Kim, R. A. King, P. Klunzinger, D. Kosenkov, T. Kowalczyk, C. M. Krauter, K. U. Lao, A. D. Laurent, K. V. Lawler, S. V. Levchenko, C. Y. Lin, F. Liu, E. Livshits, R. C. Lochan, A. Luenser, P. Manohar, S. F. Manzer, S.-P. Mao, N. Mardirossian, A. V. Marenich, S. A. Maurer, N. J. Mayhall, E. Neuscamman, C.

M. Oana, R. Olivares-Amaya, D. P. O'Neill, J. A. Parkhill, T. M. Perrine, R. Peverati, A. Prociuk, D. R. Rehn, E. Rosta, N. J. Russ, S. M. Sharada, S. Sharma, D. W. Small, A. Sodt, T. Stein, D. Stück, Y.-C. Su, A. J. W. Thom, T. Tsuchimochi, V. Vanovschi, L. Vogt, O. Vydrov, T. Wang, M. A. Watson, J. Wenzel, A. White, C. F. Williams, J. Yang, S. Yeganeh, S. R. Yost, Z.-Q. You, I. Y. Zhang, X. Zhang, Y. Zhao, B. R. Brooks, G. K. L. Chan, D. M. Chipman, C. J. Cramer, W. A. Goddard, M. S. Gordon, W. J. Hehre, A. Klamt, H. F. Schaefer, M. W. Schmidt, C. D. Sherrill, D. G. Truhlar, A. Warshel, X. Xu, A. Aspuru-Guzik, R. Baer, A. T. Bell, N. A. Besley, J.-D. Chai, A. Dreuw, B. D. Dunietz, T. R. Furlani, S. R. Gwaltney, C.-P. Hsu, Y. Jung, J. Kong, D. S. Lambrecht, W. Liang, C. Ochsenfeld, V. A. Rassolov, L. V. Slipchenko, J. E. Subotnik, T. Van Voorhis, J. M. Herbert, A. I. Krylov, P. M. W. Gill and M. Head-Gordon, Advances in molecular quantum chemistry contained in the Q-Chem 4 program package, *Mol. Phys.*, 2015, **113**, 184-215.

27. S. Grimme, J. Antony, S. Ehrlich and H. Krieg, A consistent and accurate ab initio parametrization of density functional dispersion correction (DFT-D) for the 94 elements H-Pu, *J. Chem. Phys*, 2010, **132**, 154104.
28. F. Thorning, K. Strunge, F. Jensen and P. R. Ogilby, The complex between molecular oxygen and an organic molecule: modeling optical transitions to the intermolecular charge-transfer state, *Physical Chemistry Chemical Physics*, 2021, **23**, 15038-15048.
29. R. Z. Khaliullin, E. A. Cobar, R. C. Lochan, A. T. Bell and M. Head-Gordon, Unravelling the Origin of Intermolecular Interactions Using Absolutely Localized Molecular Orbitals, *J. Phys. Chem. A*, 2007, **111**, 8753-8765.
30. P. Bultnick, R. Ponec and S. van Damme, Multicenter bond indices as a new measure of aromaticity in polycyclic aromatic hydrocarbons, *J. Phys. Org. Chem.*, 2005, **18**, 706-718.
31. R. S. Mulliken, Criteria for the Construction of Good Self-Consistent-Field Molecular Orbital Wave Functions, and the Significance of LCAO-MO Population Analysis, *J. Chem. Phys*, 1962, **36**, 3428-3439.
32. M. Mandado, M. J. Gonzalez-Moa and R. A. Mosquera, QTAIM N-center delocalization indices as descriptors of aromaticity in mono and poly heterocycles, *J Comput Chem*, 2007, **28**, 127-136.
33. B. R. Brooks, C. L. Brooks Iii, A. D. Mackerell Jr, L. Nilsson, R. J. Petrella, B. Roux, Y. Won, G. Archontis, C. Bartels, S. Boresch, A. Caflisch, L. Caves, Q. Cui, A. R. Dinner, M. Feig, S. Fischer, J. Gao, M. Hodoscek, W. Im, K. Kuczera, T. Lazaridis, J. Ma, V. Ovchinnikov, E. Paci, R. W. Pastor, C. B. Post, J. Z. Pu, M. Schaefer, B. Tidor, R. M. Venable, H. L. Woodcock, X. Wu, W. Yang, D. M. York and M. Karplus, CHARMM: The biomolecular simulation program, *J. Comput. Chem*, 2009, **30**, 1545-1614.
34. J. Huang, S. Rauscher, G. Nawrocki, T. Ran, M. Feig, B. L. de Groot, H. Grubmüller and A. D. MacKerell, CHARMM36m: an improved force field for folded and intrinsically disordered proteins, *Nat. Methods*, 2017, **14**, 71-73.
35. T. Darden, D. York and L. Pedersen, Particle mesh Ewald: An N·log(N) method for Ewald sums in large systems, *J. Chem. Phys*, 1993, **98**, 10089-10092.
36. P. Ortega, A. Zanchet, C. Sanz-Sanz, S. Gomez-Carrasco, L. Gonzalez-Sanchez and P. G. Jambrina, DpgC-Catalyzed Peroxidation of 3,5-Dihydroxyphenylacetyl-CoA (DPA-CoA): Insights into the Spin-Forbidden Transition and Charge Transfer Mechanisms*, *Chemistry*, 2021, **27**, 1700-1712.
37. J. N. Harvey, Understanding the kinetics of spin-forbidden chemical reactions, *Physical Chemistry Chemical Physics*, 2007, **9**, 331-343.

38. C. Wittig, The Landau-Zener Formula, *The Journal of Physical Chemistry B*, 2005, **109**, 8428-8430.
39. C. Zener and R. H. Fowler, Non-adiabatic crossing of energy levels, *Proceedings of the Royal Society of London. Series A, Containing Papers of a Mathematical and Physical Character*, 1932, **137**, 696-702.
40. I. Fdez Galvan, M. Vacher, A. Alavi, C. Angeli, F. Aquilante, J. Autschbach, J. J. Bao, S. I. Bokarev, N. A. Bogdanov, R. K. Carlson, L. F. Chibotaru, J. Creutzberg, N. Dattani, M. G. Delcey, S. S. Dong, A. Dreuw, L. Freitag, L. M. Frutos, L. Gagliardi, F. Gendron, A. Giussani, L. Gonzalez, G. Grell, M. Guo, C. E. Hoyer, M. Johansson, S. Keller, S. Knecht, G. Kovacevic, E. Kallman, G. Li Manni, M. Lundberg, Y. Ma, S. Mai, J. P. Malhado, P. A. Malmqvist, P. Marquetand, S. A. Mewes, J. Norell, M. Olivucci, M. Oppel, Q. M. Phung, K. Pierloot, F. Plasser, M. Reiher, A. M. Sand, I. Schapiro, P. Sharma, C. J. Stein, L. K. Sorensen, D. G. Truhlar, M. Ugandi, L. Ungur, A. Valentini, S. Vancoillie, V. Veryazov, O. Weser, T. A. Wesolowski, P. O. Widmark, S. Wouters, A. Zech, J. P. Zobel and R. Lindh, OpenMolcas: From Source Code to Insight, *J Chem Theory Comput*, 2019, **15**, 5925-5964.
41. B. F. Minaev and R. R. Valiev, Spin-Orbit coupling effects in O2 activation by cofactor-independent 2,4 dioxygenase, *Ukr. Biochem. J.*, 2019, **91**, 38.
42. P. J. Silva, Refining the reaction mechanism of O(2) towards its co-substrate in cofactor-free dioxygenases, *Peerj*, 2016, **4**, e2805-e2805.
43. G. Knizia, Intrinsic Atomic Orbitals: An Unbiased Bridge between Quantum Theory and Chemical Concepts, *Journal of Chemical Theory and Computation*, 2013, **9**, 4834-4843.
